# Memory-like Liver Natural Killer Cells are Responsible for Islet Destruction in Secondary Islet Transplantation

**DOI:** 10.1038/s41598-018-37395-9

**Published:** 2019-01-31

**Authors:** Y. Saeki, K. Ishiyama, N. Ishida, Y. Tanaka, H. Ohdan

**Affiliations:** 1 Department of Gastroenterological and Transplant Surgery, Applied Life Sciences, Institute of Biomedical & Health Science, Hiroshima University, Hiroshima, Japan; 2 Department of Surgery, National Hospital Organization Kure Medical Center and Chugoku Cancer Center, Hiroshima, Japan

## Abstract

We previously demonstrated the pivotal role of natural killer (NK) cells in islet graft loss during the early phase after intraportal syngeneic islet transplantation (IT). Liver-resident DX5^−^ NK cells were reported to possess memory-like properties, distinguishing them from conventional DX5^+^ NK cells. Here, we investigated the impact of primary IT-induced liver DX5^−^ NK cells on the engraftment of secondary-transplanted islets in mice. The culture of liver NK cells isolated from naive mice with TNF-α, IFN-γ, and IL-lβ, mimicking instant blood-mediated inflammatory reaction, led to significantly increased DX5^−^ NK cell percentage among total liver NK cells. Consistently, the prolonged expansion of DX5^−^ CD69^+^ TRAIL^+^ CXCR3^+^ NK cells was observed after intraportal IT of 300 syngeneic islets (marginal mass). In most diabetic mice, 400 syngeneic islets of primary IT were sufficient to achieve normoglycaemia, whereas the same mass after secondary IT failed to induce normoglycaemia in mice that received 200 syngeneic islets during primary IT. These findings indicated that liver-resident DX5^−^ NK cells significantly expanded even after syngeneic IT, and that these memory-like NK cells may target both originally engrafted and secondary-transplanted islets. Furthermore, anti-TNF-α treatment suppressed the expansion of liver-resident DX5^−^ NK cells, resulting in successful islet engraftment after sequential ITs.

## Introduction

Clinical outcome of islet transplantation (IT) is becoming comparable to that of pancreas transplantation for a subgroup of patients with type 1 diabetes mellitus^1–4^. However, multiple ITs are required for competent long-term clinical outcomes, because islet grafts undergo rapid reduction following intraportal infusion owing to embolism-induced ischemic injury, antigen-nonspecific inflammatory events, and other processes^5–12^. To achieve successful IT, several investigators have questioned the suitability of the liver as the appropriate site for islet graft survival^6,13,14^. Immunologically, innate inflammatory response, designated as instant blood-mediated inflammatory reaction (IBMIR), was suggested to represent the main cause of islet destruction^8,15,16^. Macrophages and natural killer (NK) T-cells are also believed to play a key role in the early inflammatory events that adversely affect islet engraftment^7,11^. Furthermore, we have reported that liver mononuclear cells (LMNCs) contain a large population of NK cells, which possess increased cytotoxic activity in comparison with peripheral blood NK cells^17–21^. Both TNF-related apoptosis-inducing ligand (TRAIL) expression on liver NK cells and their cytotoxicity against syngeneic and allogeneic islets significantly increased following intraportal IT^6^. Liver NK cell cytotoxicity against islets was partially but significantly inhibited by adding anti-TRAIL mAb. These results suggested that liver NK cells play a pivotal role in the destruction of islets transplanted into the liver in mouse models.

NK cells represent a part of the innate immune system, and they are important effectors activated during the host innate immune response to intracellular pathogens and for tumour immunosurveillance^22,23^. NK cells are classically believed unable to differentiate into memory cells. Immunological memory, the ability to remember a previous encounter with an antigen and provide an enhanced response upon secondary encounter with the same antigen, has been considered the hallmark of T- and B-cells belonging to the adaptive immune system^24–26^. Furthermore, memory cells are long-lived and phenotypically distinct from their naive counterparts^24^. Accumulating evidence suggests that NK cells also exhibit memory properties and are divided into several subsets according to the nature of their inducers^24,27–30^. Specifically, liver-resident NK cells lack DX5, the α2 integrin chain CD49b (a classical NK cell marker), and express TRAIL^29^. These DX5^−^ NK cells are involved in the immunological memory response and their hematopoietic progenitor and precursor cells can be found in the liver^29^.

Several investigators reported that immune cells are involved in islet destruction^7,11,31^; however, few studies have investigated multiple ITs using clinically relevant approaches in a mouse model, and the immune status following multiple ITs is not well characterised. Therefore, to evaluate the mechanism of NK cell activation, we investigated the involvement of liver-resident DX5^−^ NK cells in islet destruction in both early and late phases after intraportal ITs. Furthermore, we developed an *in vivo* model, which allowed us to compare the outcomes of the primary and secondary syngeneic ITs, and investigated the effects of the primary intraportal IT on the secondary IT by defining the population dynamics of liver resident DX5^−^ memory-like NK cells.

## Results

### Naive liver DX5^−^ NK cells express CD69, TRAIL, and CXCR3, which target islet grafts

**Figure 1:**
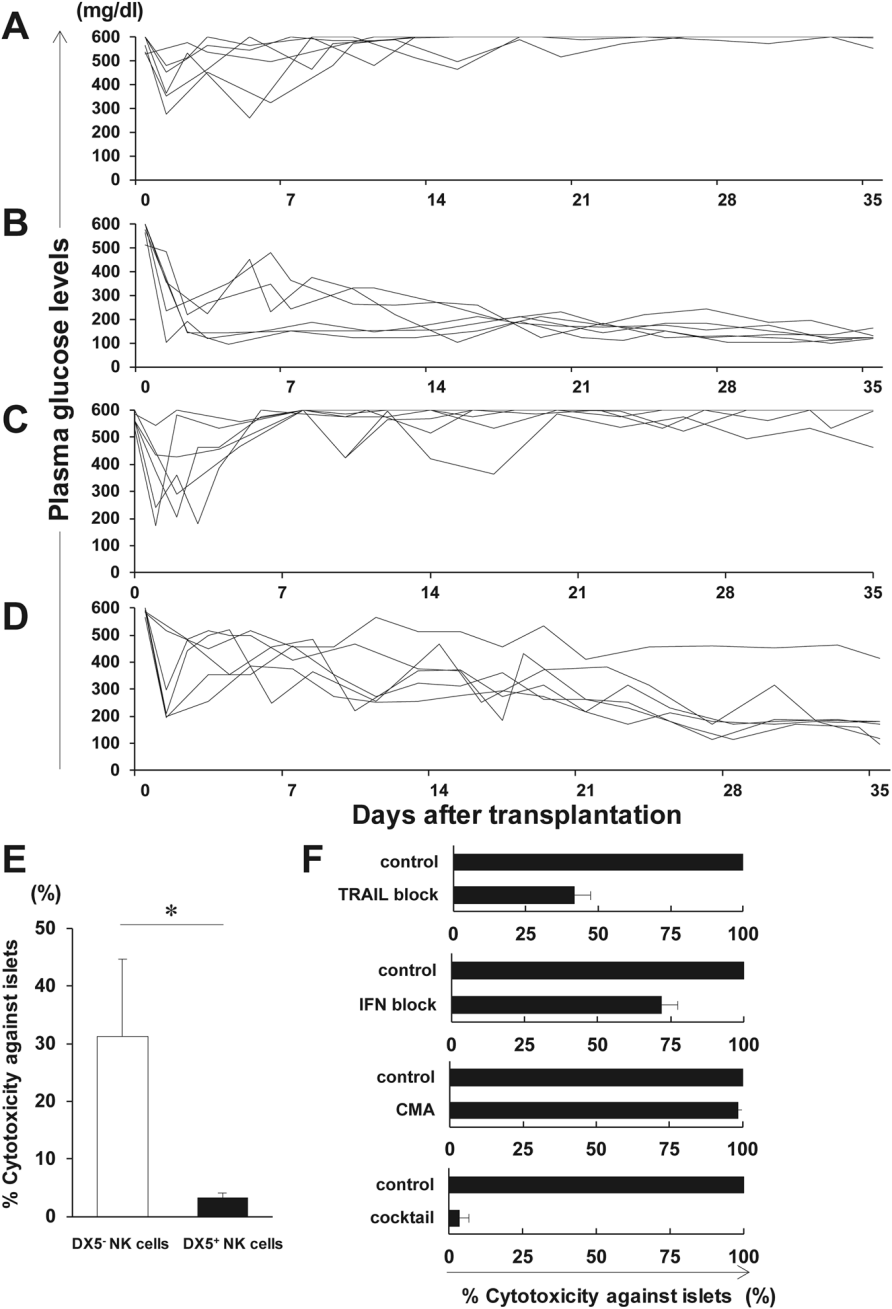
Involvement of liver DX5^−^ natural killer (NK) cells in islet destruction after islet transplantation (IT). (**A**,**B**) Syngeneic islet graft survival in diabetic C57BL/6 (B6) *wild-type (WT)* or B6 *CXCR3*^−/−^ mice was monitored by measuring the non-fasting plasma glucose level after intraportal IT. Glucose levels less than 200 mg/dL indicated diabetes reversal. Blood glucose levels of the control diabetic B6 *WT* or B6 *CXCR3*^−/−^ mice that were intraportally transplanted with 200 syngeneic islets (n = 6). Data were collected from 5 independent experiments. (**C,D**) Syngeneic islet graft survival in diabetic B6 *WT* or B6 *Tbx21*^−/−^ mice was monitored by measuring the non-fasting plasma glucose level after intraportal IT. Glucose levels less than 200 mg/dL indicated diabetes reversal. Blood glucose levels of the control diabetic B6 *WT* or B6 *Tbx21*^−/−^ mice that were intraportally transplanted with 200 syngeneic islets (n = 6). Data were collected from 4 independent experiments. (**E**) The cytotoxicity of NK cells from B6 mice was tested using islets isolated from B6 mice as targets. NK cells were isolated from the liver of naive B6 mice and were used as effector cells at an E:T ratio of 10^5^:1. The cytotoxicity of NK cells is shown in bar graphs (DX5^−^ NK cells, open bar; DX5^+^ NK cells, solid bar) as the mean ± SD of 3 independent experiments (n = 3, pooled liver mononuclear cells were from 18 mice per experiment). ^*^p < 0.05. (**F**) The cytotoxicity of DX5^−^ NK cells with or without anti-TRAIL mAb, anti-IFN-γ mAb, or concanamycin A (CMA) is shown in bar graphs as the mean ± SD (n = 3 per mAb). The experiments were repeated 12 times (n = 17 mice per experiment). Data were collected from 7 independent experiments.

MNCs were isolated from the livers or spleens of naive B6 mice. As previously reported, liver NK cells contained numerous DX5^−^ NK cells compared to splenic NK cells (p < 0.001) (Supplementary Fig. S1)^29,32^. CD69, TRAIL, and CXCR3 expression on liver DX5^−^ NK cells was significantly higher than that on DX5^+^ NK cells (p < 0.001, for all) (Supplementary Fig. S1)^32^. CD69 is known as an early activation marker induced in NK, T, and B cells in response to inflammatory stimuli^33^. TRAIL has already been shown to induce apoptosis through binding its respective receptors, death receptor (DR) 4 and DR5^34^. We have previously confirmed that dissociated islets express the TRAIL receptor DR5^6^. It has been reported that CXCL10 secreted from β cells activates and attracts autoreactive T cells and macrophages to the islets via CXCR3 after viral infection in human autoimmune type 1 diabetes^35–37^. All these findings, together with the remarkable expression of CXCR3 on DX5^−^ NK cells, demonstrate the possibility that the chemotaxis of liver resident NK cells, induced by the CXCL10-CXCR3 pathway, contributes to islet graft survival after IT. To address this issue, we further investigated the role of the CXCR3 molecule in IT. We used B6 *wild-type* (WT) or B6 *CXCR3*^−/−^ mice. In *in vitro* transwell migration assays, liver NK cells from WT compared with *CXCR3*^−/−^ mice displayed significantly increased migration to islets (p = 0.015) (Supplementary Fig. S2). In B6 WT recipients of 200 islets, hyperglycaemia could not be ameliorated after IT (Fig. 1A). However, normoglycaemia was achieved in B6 *CXCR3*^−/−^ recipients of the same dose of islets (Fig. 1B). A significant difference in the rate of normoglycaemia achievement in B6 WT mice (0%, 0/6) or B6 *CXCR3*^−/−^ mice (100%, 6/6) was observed (p = 0.0022).

We next investigated the cytotoxicity of liver DX5^−^ NK cells against islets. Cytotoxicity assays revealed that the isolated DX5^−^ exhibited a higher cytotoxicity against islets than DX5^+^ NK cells, even in the steady state (Fig. 1E). The cytotoxicity of DX5^−^ NK cells against islets was partially inhibited by either anti-TRAIL mAb or anti-IFN-γ mAb, but not by concanamycin A (CMA) (Fig. 1F). The cytotoxicity was almost completely inhibited by the combination of the antibodies. To investigate the direct evidence of the involvement of liver DX5^−^ NK cells in islet destruction, we first attempted to establish an experimental model to evaluate islet engraftment in B6 *Rag-2*^−/−^ *γ chain*^−/−^ mice, which completely lack T, B, NKT, and NK cells, reconstructed with the liver resident NK cells. On day 14 after adoptive transfer of the total liver NK cells containing both DX5^−^ and DX5^+^ NK cells, DX5^−^ NK cells were predominantly engrafted in the liver (p < 0.000001) (Supplementary Fig. S3). The expression of CD69, TRAIL, and CXCR3 on liver NK cells in the recipients considerably increased compared with that of those on transferred NK cells (p < 0.000001, p < 0.000001, p < 0.01, respectively) (Supplementary Fig. S3). These data were consistent with a previous report that DX5^−^ NK cells preferentially traffic to the liver^29^. Hence, it is less feasible to accurately compare islet engraftment in the liver between DX5^−^ NK- and DX5^+^ NK cell-transferred hosts owing to their differences in chemotactic responses and the spontaneous activation of NK cells in consequence of adoptive transfer. Instead of an adoptive transfer study, we employed *Tbx21*^−/−^ mice lacking immature TRAIL^+^ DX5^−^ NK cells^32^. In B6 *Tbx21*^−/−^ recipients of 200 islets, normoglycaemia was almost achieved (83%, 5/6), whereas hyperglycaemia could not be ameliorated after IT in B6 WT recipients of the same dose of islets (0%, 0/6) (p = 0.0152) (Fig. 1C,D). Taken together, our findings suggest that liver DX5^−^ NK cells have cytotoxic activity against islets via the TRAIL-TRAIL DR5 and CXCR3-CXCL10 pathways.

### IBMIR induces the expansion of liver DX5^−^ NK cells *in vitro*

**Figure 2:**
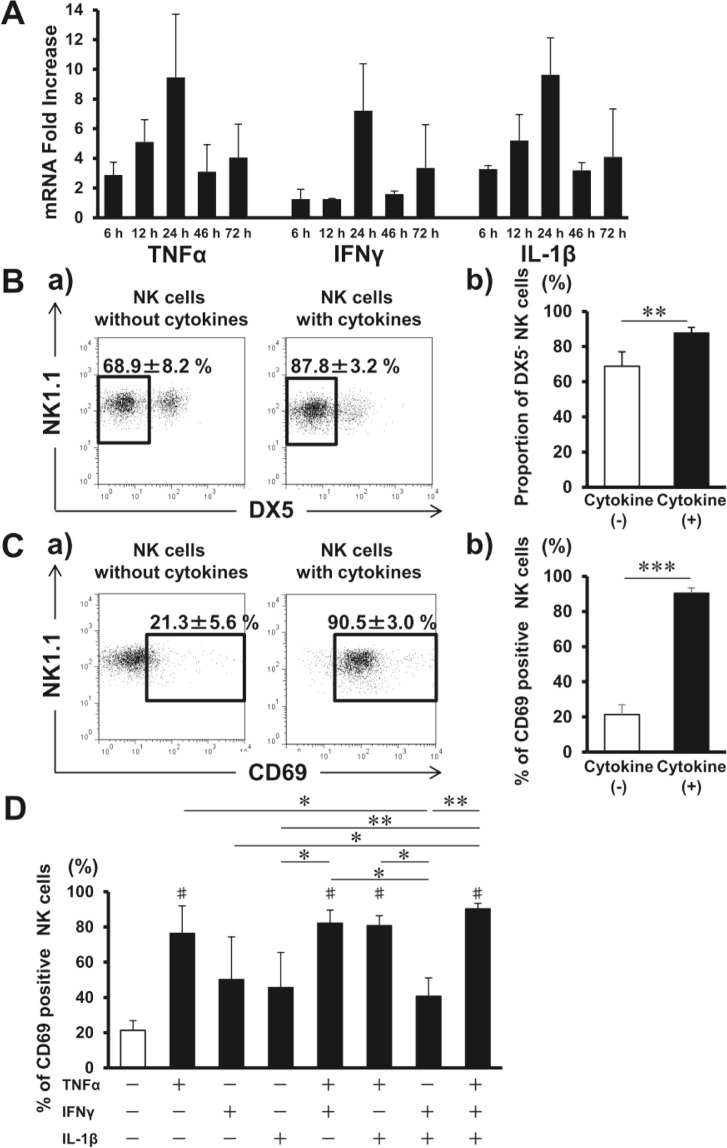
Liver natural killer (NK) cells are activated during instant blood-mediated inflammatory reaction (IBMIR). (**A**) Livers were harvested from C57BL/6J (B6) mice that received 300 syngeneic islets 6, 12, 24, 48, and 72 h after islet transplantation. Time course of intrahepatic mRNA for TNF-α, IFN-γ, and IL-lβ in islet transplant recipients after transplantation were compared with that of non-treated *wild-type* B6 mice (naive livers) as quantified by real-time RT-PCR. The relative fold increase was calculated using the delta-delta Ct method. Data are presented as the means ± standard deviation (SD) (n = 3–4). (**B**) Liver TCRβ^−^ NK1.1^+^ NK cells were separated from liver mononuclear cells and cultured without or with TNF-α, IFN-γ, and IL-1β. Cells were harvested after 24 h and analysed with flow cytometry. Representative flow cytometry plots of NK1.1 and DX5 in isolated NK cells after the incubation without or with three cytokines. The proportions of DX5^−^ NK cells among total NK cells are shown (n = 5). The data in bar graphs are presented as the means ± SD of 4 independent experiments. ^**^p < 0.01. (**C**) Representative flow cytometry plots of NK1.1 and CD69 in isolated NK cells after their incubation without or with three cytokines. The percentages of cells expressing CD69 among total liver NK cells are shown in bar graphs as the means ± SD of 4 independent experiments (n = 5). ^***^p < 0.001. (**D**) Liver NK cells treated with the cytokine combinations for 24 h (n = 4–5). The data in bar graphs are presented as the means ± SD of 4 independent experiments. ^*^p < 0.05; ^**^p < 0.01. ^#^p < 0.001, compared with the results of liver NK cells without cytokines.

Consistent with predominant and transient TNF-α, IFN-γ, and IL-1β production during IBMIR^8,12^, such cytokine mRNA expression significantly increased in the liver with peak value at 24 h after syngeneic IT (Fig. 2A). Serum TNF-α was detected in mouse after IT as well (Supplementary Fig. S4). To investigate the possibility that IBMIR triggers NK cell activation, liver NK cells from B6 mice were cultured for 24 h in the presence of the three cytokines. The proportion of DX5^−^ NK cells among total NK cells increased after the incubation (p < 0.01) (Fig. 2B), and CD69 expression on liver NK cells considerably increased after the culture (p < 0.001) (Fig. 2C). Liver NK cell CD69 mean fluorescence intensity (MFI) also increased following the incubation (without *vs*. with cytokines; 6.8 ± 1.3 *vs*. 68.7 ± 9.4, p < 0.01, not shown in Figure). To define the predominant cytokine activating NK cells, each cytokine was added to or removed from the culture. CD69-expressing NK cell proportion increased when cultured with TNF-α alone, but not with either IFN-γ or IL-1β (Fig. 2D). Thus, TNF-α was the predominant cytokine activating DX5^−^ NK cells which express both TNF-α 1 and 2 receptors (Supplementary Fig. S5). When combined with the other two cytokines, the absolute DX5^−^ NK cell number further increased (Supplementary Fig. S6).

### Proportion of DX5^−^ NK cells increases early after intraportal IT

**Figure 3:**
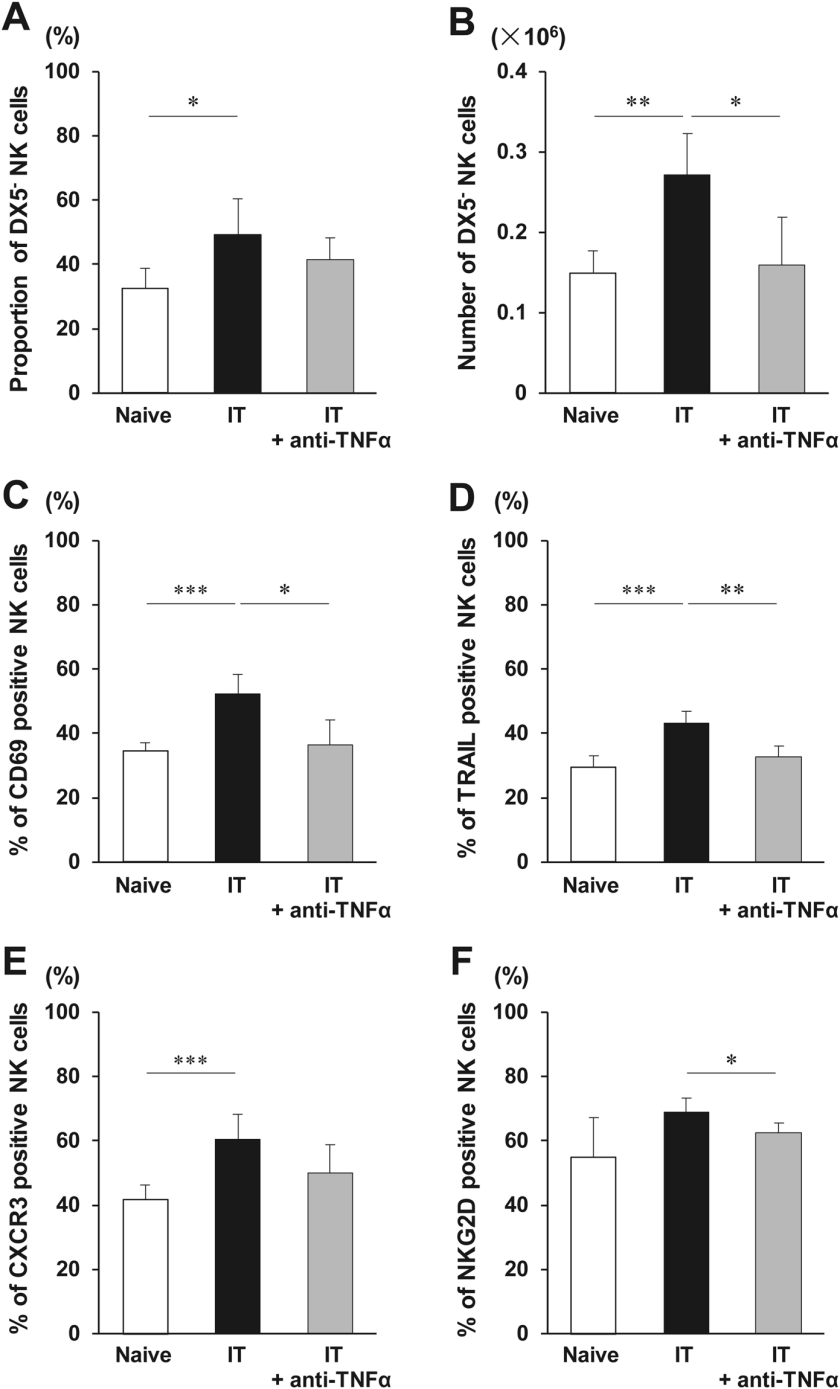
Phenotypic alterations of liver natural killer (NK) cells early after islet transplantation (IT). C57BL/6 *wild-type* mice administered PBS or anti-TNF-α antibody were treated with 300 syngeneic islets. Phenotypic alterations of NK cells in the liver were analysed 24 h after intraportal IT. (**A**,**B**) Proportion of TCRβ^−^ NK1.1^+^ DX5^−^ NK cells in total NK cells and the absolute number of DX5^−^ NK cells obtained from the liver of mice that received 300 syngeneic islets, and that were treated or not with anti-TNF-α antibody (naive group, open bar; group that received transplantation, solid bar; group that received islet and anti-TNF-α antibody treatment, gray bar) (n = 5–7). The data in bar graphs are shown as the means ± standard deviation (SD) of 5 independent experiments. ^*^p < 0.05. ^**^p < 0.01. (**C**–**F**) Percentages of CD69-, TRAIL-, CXCR3-, or NKG2D positive NK cells in the liver after intraportal IT were analysed with flow cytometry (naive group, open bar; group that received transplantation, solid bar; group that received islet and anti-TNF-α antibody treatment, gray bar) (n = 5–7). The data in bar graphs are shown as the means ± SD of 5 independent experiments. ^*^p < 0.05. ^**^p < 0.01. ^***^p < 0.001.

To investigate the role of TNF-α in NK cell activation, LMNCs were collected 24 h after IT in mice treated with/without anti-TNF-α antibody. The NK cell proportion in LMNCs significantly increased 24 h after IT, compared with that in non-transplanted naive mice (9.5 ± 0.6% *vs*. 8.1 ± 0.9%, respectively, p = 0.022, not shown in Figure). The absolute number of NK cells obtained from the whole liver tended to increase 24 h after IT, compared with that in the nontransplanted naive mice (0.523 ± 0.068 × 10^6^ *vs*. 0.463 ± 0.055 × 10^6^, respectively, p = 0.210, not shown in Figure). The proportion of DX5^−^ NK cells in total NK cells and the number of DX5^−^ NK cells increased 24 h after IT (p = 0.013, p = 0.009, respectively) (Fig. 3A,B). Based on FCM histogram, DX5^−^ NK cells were clearly distinguished from DX5^+^ NK cells by the MFI value of DX5 expression even after IT (Supplementary Fig. S7), suggesting that the increase of DX5^−^ NK cells was not consequent to reduced DX5^+^ NK cell DX5 expression. In contrast, the number of DX5^+^ NK cells did not increase 24 h after IT, compared with that in the nontransplanted naive mice (0.268 ± 0.085 × 10^6^ *vs*. 0.315 ± 0.067 × 10^6^, respectively, p = 0.411, not shown in Figure). IT increased the expression of CD69 and TRAIL on liver NK cells, although it did not change their expression on either DX5^−^ and DX5^+^ NK cells (Supplementary Figs S8 and 9). The treatment with the TNF-α-neutralising antibody prior to IT significantly inhibited the changes in DX5^−^ NK cell number (p = 0.048) (Fig. 3B). Similarly, the increase in CD69 and TRAIL expression on liver NK cells in IT recipients was inhibited by anti-TNF-α antibody as well (p = 0.012, p = 0.003, respectively) (Fig. 3C,D). Anti-TNF-α antibody of IT recipients led to the reduction in the expression of CXCR3 and NKG2D on liver NK cells, although the former level did not reach statistical significance (p = 0.108, p = 0.037, respectively) (Fig. 3E,F). Thus, TNF-α had key roles in DX5^−^ NK cell expansion early after IT even in an *in vivo* model. To investigate the difference in NK cell activation between syngeneic and allogeneic IT, LMNCs were also collected 24 h after IT in mice treated with allogeneic islets. The liver NK cell activation did not significantly differ between syngeneic and allogeneic IT (Supplementary Fig. S10).

### Conversion of DX5^+^ NK cells to a DX5^−^ phenotype was not observed after IT

**Figure 4:**
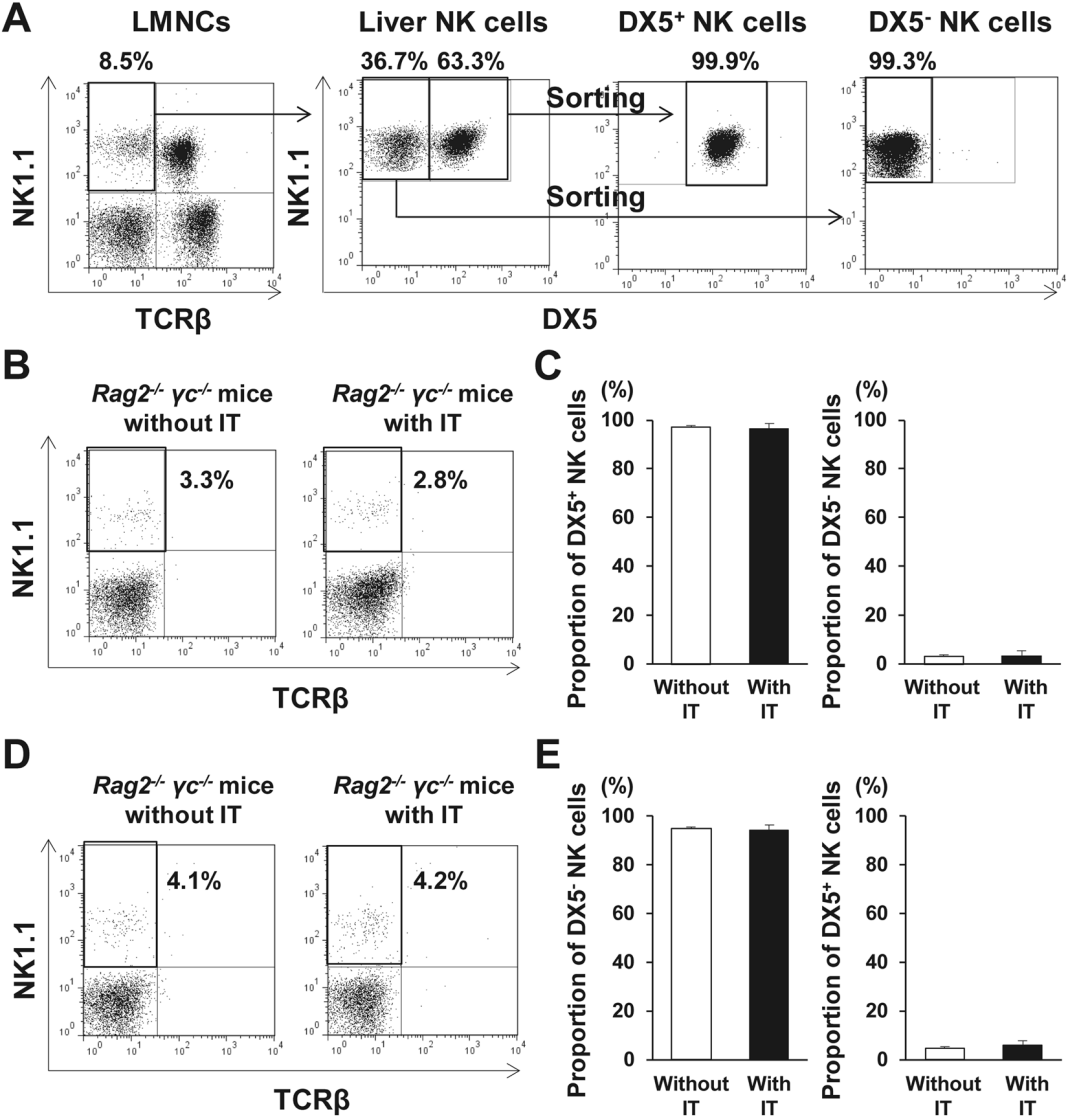
Propagation of liver DX5^−^ natural killer (NK) cells in response to islet transplantation (IT). (**A**) TCRβ^−^ NK1.1^+^ DX5^+^ NK cells and DX5^−^ NK cells were isolated from liver mononuclear cells (LMNCs) of C57BL/6 (B6) *wild-type* mice. Representative dot plots show the isolated DX5^+^ NK cells and DX5^−^ NK cells. (**B**) Isolated DX5^+^ NK cells were transferred into B6 *Rag-2*^−/−^ *γ chain*^−/−^ mice, which was followed by IT, after which LMNCs from the recipient mice were analysed. Representative dot plots show the gated TCRβ^−^ NK1.1^+^ NK cells and their percentage in total liver NK cells from DX5^+^ NK cell-transferred recipients with/without IT. (**C**) Bar graph, representing the mean percentage ± standard deviation (SD) of each subset of NK cells in total NK cells isolated from B6 *Rag-2*^−/−^ *γ chain*^−/−^ mice that received DX5^+^ NK cells, followed or not by IT (n = 5). Data were collected from of 2 independent experiments. (**D**) Isolated DX5^−^ NK cells were transferred into B6 *Rag-2*^−/−^ *γ chain*^−/−^ mice, which was followed by IT, after which LMNCs from the recipient mice were analysed. Representative dot plots show the gated TCRβ^−^ NK1.1^+^ NK cells and their percentage in total liver NK cells from DX5^−^ NK cell-transferred recipient with/without IT. (**E**) Bar graph, representing the mean percentage ± SD of each subset of NK cells in total NK cells isolated from B6 *Rag-2*^−/−^ *γ chain*^−/−^ mice that received DX5^−^ NK cells, followed or not by IT (n = 5). Data were collected from of 2 independent experiments.

One possible explanation for the increase of DX5^−^ NK cell proportion after IT is the direct expansion of DX5^−^ NK cells during IBMIR, or, alternatively, DX5^−^ NK cell population may arise from DX5^+^ NK cells in the presence of TNF-α. To elucidate this issue, liver DX5^+^ NK cells (purity >93%; Fig. 4A) were adoptively transferred into B6 *Rag-2*^−/−^ *γ chain*^−/−^ mice that received IT. The transferred DX5^+^ NK cells were detected in the liver of the recipient B6 *Rag-2*^−/−^ *γ chain*^−/−^ mice (Fig. 4B). Even after IT, the DX5^+^ NK cell proportion in LMNCs was constant, whereas DX5^−^ NK cells remained undetectable (Fig. 4C). As the counterpart, liver DX5^−^ NK cells were also adoptively transferred into B6 *Rag-2*^−/−^ *γ chain*^−/−^ mice that received IT. In those mice, only DX5^−^ NK cells were detected even after IT as expected (Fig. 4D,E). Thus, conversion of DX5^+^ NK cells to a DX5^-^ phenotype was not observed after IT, speculating an alternative explanation, i.e. direct expansion of DX5^−^ NK cells. This speculation was supported by higher DX5^−^ NK cell numbers in the transferred mice with IT than in those without IT, although the difference did not reach a statistical significance (Supplementary Fig. S11).

The expansion of DX5^−^ NK cells after IT, observed in the WT mice described above (Fig. 3), might be due to the proliferation of these cells. To examine the proliferative potential of liver NK cells after IT, Ki-67 expression on liver DX5^−^ and DX5^+^ NK cells was assessed. Ki-67 expression on both liver DX5^−^ and DX5^+^ NK cells of mice that received islets did not differ from that on both liver DX5^−^ and DX5^+^ NK cells of naive mice, suggesting that the expansion of DX5^−^ NK cells was not consequent to the proliferation of DX5^−^ NK cells, potentially due to the recruitment of these cells/precursors from other sites (Supplementary Fig. S12).

### Intraportal IT consistently sustains the activation of liver DX5^−^ NK cells

**Figure 5:**
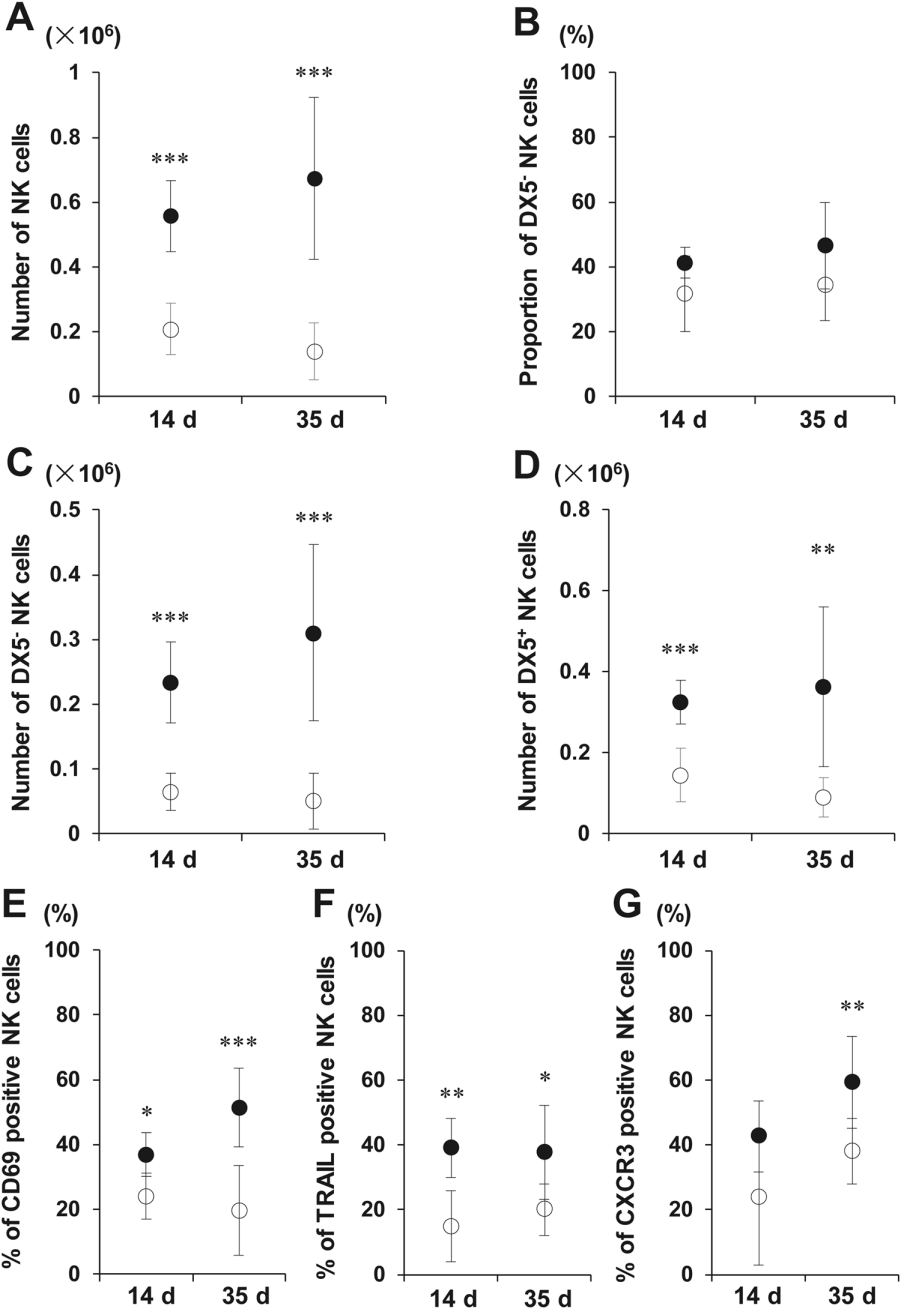
Expansion of liver DX5^−^ natural killer (NK) cells at the late phase after islet transplantation (IT). Phenotypic alteration of TCRβ^−^ NK1.1^+^ NK cells in the liver from diabetic C57BL/6 (B6) mice was analysed 14 days (n = 6, 3 independent experiments) or 35 days (n = 8, 6 independent experiments) after intraportal IT of syngeneic 300 islets. The data from transplanted diabetic mice are compared with the data from control diabetic B6 mice (open circles; control diabetic B6 mice, closed circles; transplanted diabetic mice). Diabetic B6 mice treated with streptozotocin 7 days before were used as recipients. (**A**) The absolute number of NK cells obtained from the whole livers of diabetic mice that were transplanted with 300 islets. ^***^p < 0.001, compared with control. (**B**,**C**) Proportion of DX5^−^ NK cells in total NK cells and the absolute number of DX5^−^ NK cells obtained from the whole liver of diabetic mice that were transplanted with 300 islets. ^***^p < 0.001, compared with control. (**D**) The absolute number of DX5^+^ NK cells obtained from the whole liver of diabetic mice that were transplanted with 300 islets. ^**^p < 0.01; ^***^p < 0.001, compared with control. (**E**–**G**) The proportion of liver NK cells positive for markers at the indicated time points. ^*^p < 0.05; ^**^p < 0.01; ^***^p < 0.001, compared with control.

As an additional clinically relevant model of islet engraftment, we performed IT of 300 syngeneic islets into STZ-induced diabetic mice. LMNCs isolated from the islet recipients were obtained at 14 and 35 days after IT. LMNCs from control diabetic mice who did not receive islets were obtained at the corresponding time points. NK cell numbers at both time points were significantly higher than those from non-IT controls (all p < 0.001) (Fig. 5A); the numbers of both subsets of NK cells were significantly higher than those from non-IT controls (DX5^−^ NK cells: p < 0.001, p < 0.001; DX5^+^ NK cells: p < 0.001, p < 0.01 for days 14 and 35, respectively) (Fig. 5C,D), although the DX5^−^ NK cell proportion in total NK cells of the islet recipients showed a tendency to increase (p = 0.119 at day 14, p = 0.086 at day 35) (Fig. 5B). Among liver NK cells, the CD69-, TRAIL-, and CXCR3-positive cell proportions in the IT recipients were significantly higher than those in the non-IT control (CD69: p = 0.017, p < 0.001; TRAIL: p = 0.004, p = 0.013; CXCR3: p = 0.136, p = 0.009 for days 14 and 35, respectively) (Fig. 5E–G). CD69-, TRAIL-, and CXCR3-positive DX5^−^ NK cell proportions in the IT recipients were also significantly higher (Supplementary Fig. S13). Thus, intraportal IT consistently sustained liver NK cell activation.

### Primary IT inhibits the reversal of diabetes in recipients with the sufficient mass of secondary transplanted islets

**Figure 6:**
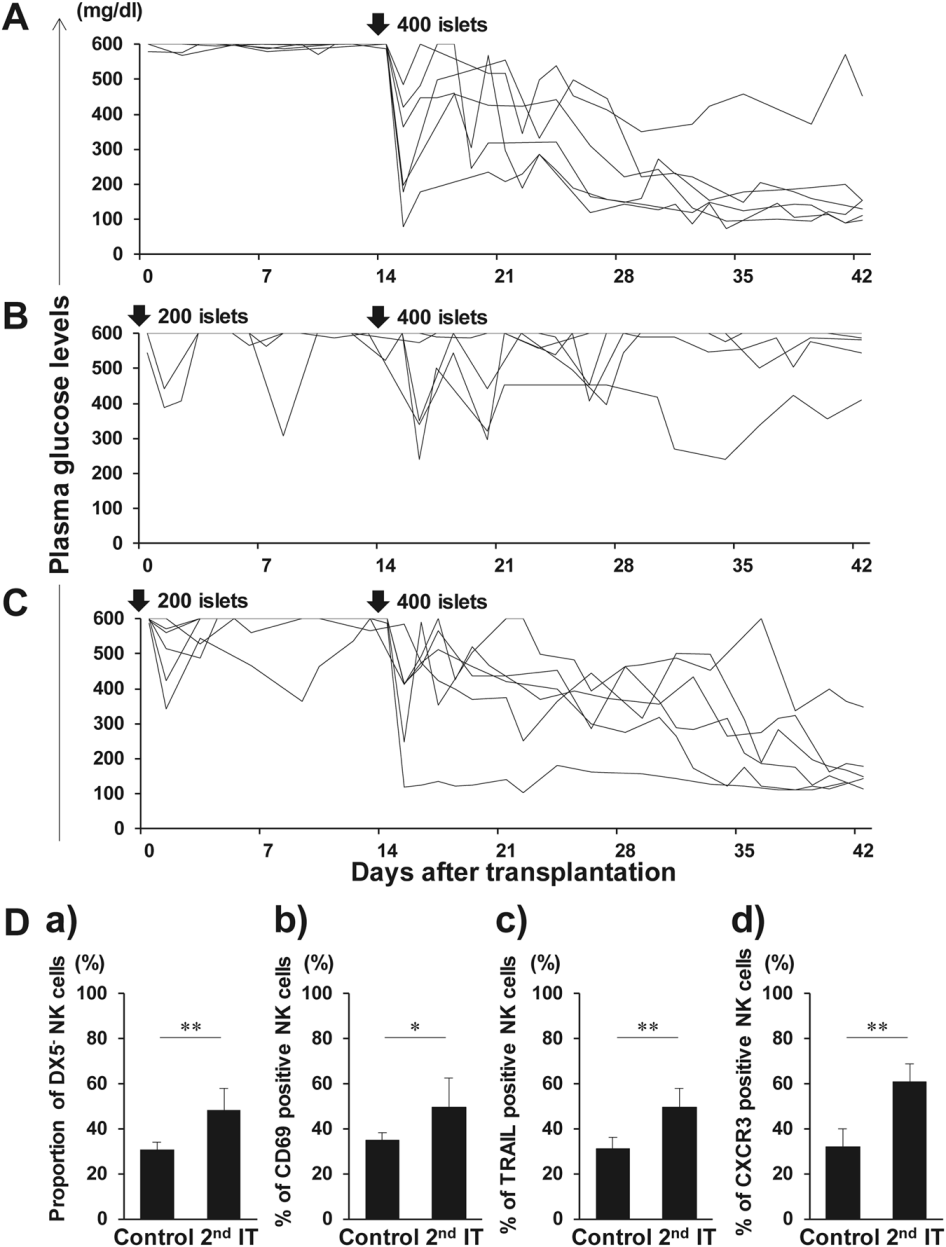
Graft survival after secondary islet transplantation (IT). Syngeneic islet graft survival in diabetic C57BL/6 (B6) mice was monitored by measuring the non-fasting plasma glucose level after intraportal IT. Glucose levels less than 200 mg/dL indicated diabetes reversal. Liver TCRβ^−^ NK1.1^+^ natural killer (NK) cells were harvested at day 42, and analysed with flow cytometry. (**A**) Blood glucose levels of the control diabetic B6 mice that were intraportally transplanted with 400 syngeneic islets (n = 6). Data were collected from 4 independent experiments. (**B**) Blood glucose levels of diabetic B6 mice that were intraportally transplanted with 400 syngeneic islets 14 days after the primary transplantation of 200 syngeneic islets (n = 6). Data were collected from 4 independent experiments. (**C**) Blood glucose levels of anti-asialo GM1-treated diabetic B6 mice that were intraportally transplanted with 400 syngeneic islets 14 days after the primary transplantation of 200 syngeneic islets (n = 6). Diabetic B6 mice were treated with intraperitoneal injection of rabbit anti-asialo GM1 serum on days −3, 0, and 14 of the secondary IT. Data were collected from 4 independent experiments. (**D**) Proportion of DX5^−^ NK cells in total NK cells obtained from the control and secondary IT mice. Expression of CD69, TRAIL, and CXCR3 on these cells was analysed. Data are presented as the means ± standard deviation of 4 independent experiments. ^*^p < 0.05; ^**^p < 0.01.

The sustained activation of liver DX5^−^ NK cells after the primary IT might interfere the engraftment of the secondary transplanted islets. We have previously shown that NK cell depletion leads to successful transplantation of 200 islets in STZ-induced diabetic *CD1d*^−/−^ mice, indicating that liver NK cells are involved in islet destruction during transplantation of 200 islets^38^. Hence, 200 islets were used as priming dose during the IT to elucidate whether the activation of liver NK cells affects the engraftment of 400 secondary transplanted islets. In the control group that did not receive the priming dose, normoglycemia was nearly achieved in the recipients following the transplantation of 400 islets (Fig. 6A). However, hyperglycaemia could not be ameliorated in the recipients that received 400 islets during the secondary IT (Fig. 6B). A significant difference in the rate of normoglycemia achievement in diabetic recipient mice after the primary 400 IT (83%, 5/6) or the secondary 400 IT after priming (0%, 0/6) was observed (p = 0.015). In comparison with that in the control group, the proportion of liver DX5^−^ NK cells expressing CD69, TRAIL, and CXCR3 significantly increased after secondary IT (p = 0.008) (Fig. 6D). Administration of ASGM1 antibody, which significantly and exclusively reduced NK cells but barely influenced NKT cells (Supplementary Fig. S14), improved the 400 secondary transplanted islet engraftment (83%, 5/6) (Fig. 6C), compared with that in the transplanted mice without antibody (0%, 0/6) (Fig. 6B) (p = 0.015). Thus, liver NK cells play a significant role in secondary islet rejection.

### Anti-TNF-α antibody regulates liver DX5^−^ NK cell expansion after secondary IT leading to enhanced secondary transplanted islet engraftment

**Figure 7:**
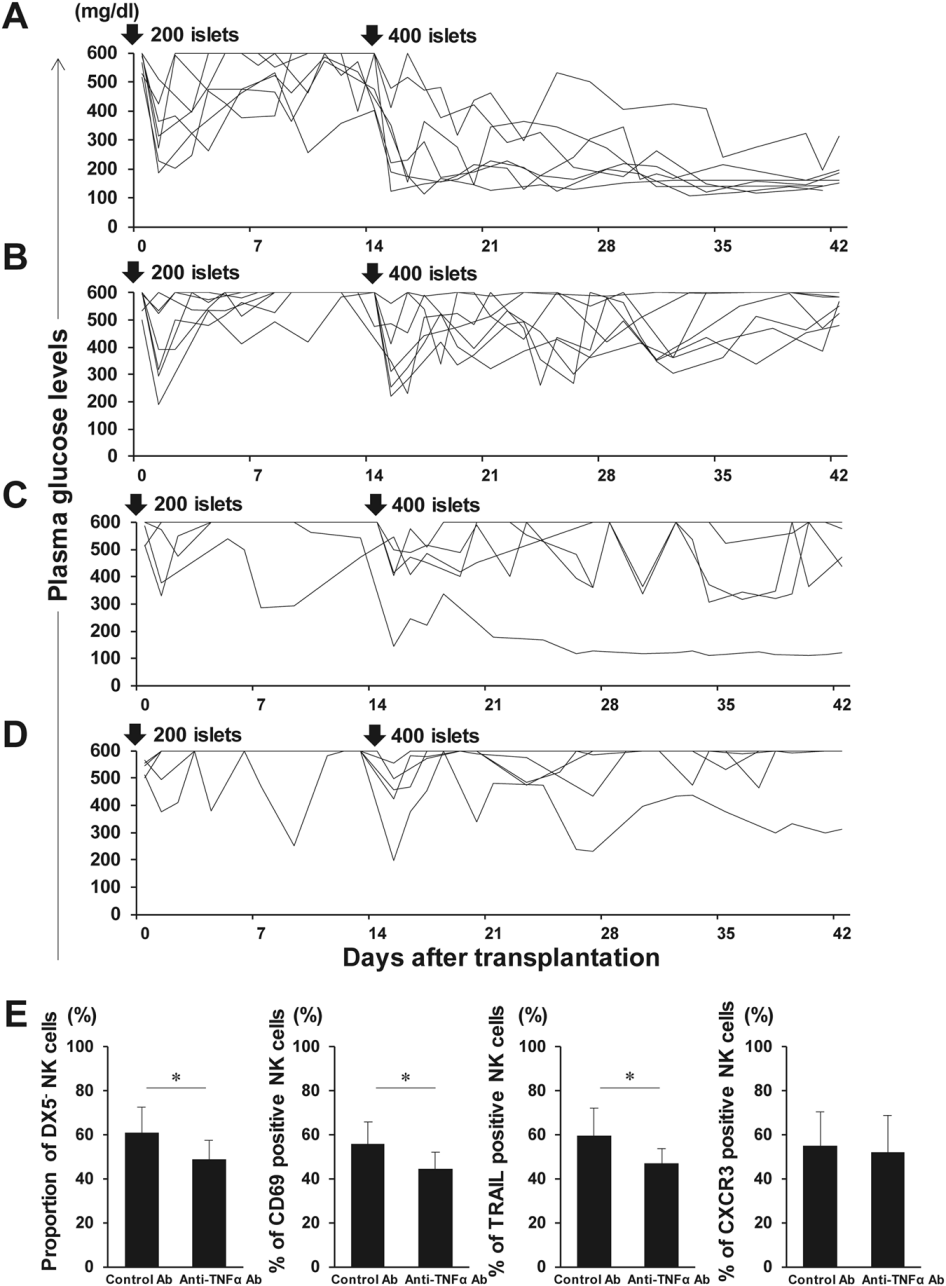
Graft survival after secondary islet transplantation (IT) with anti-TNF-α antibodies. Syngeneic islet graft survival in diabetic C57BL/6 (B6) mice that were intraportally transplanted with 400 syngeneic islets 14 days after the primary transplantation of 200 syngeneic islets was monitored by measuring the non-fasting plasma glucose level after intraportal IT. Glucose levels less than 200 mg/dL indicated diabetes reversal. Liver TCRβ^−^ NK1.1^+^ natural killer (NK) cells were harvested at day 42 and analysed with flow cytometry. (**A**) Diabetic B6 mice were treated with intraperitoneal injection of anti-TNF-α antibodies on days 0, 3, 7, and 10 of each IT (n = 7). Data were collected from 5 independent experiments. (**B**) Diabetic B6 mice were treated with intraperitoneal injection of normal goat IgG antibodies on days 0, 3, 7, and 10 of each IT (n = 7). Data were collected from 5 independent experiments. (**C**) Diabetic B6 mice were treated with intraperitoneal injection of anti-TNF-α antibodies on days 0, 3, 7, and 10 of the secondary IT (n = 5). Data were collected from 4 independent experiments. (**D**) Diabetic B6 mice were treated with intraperitoneal injection of normal goat IgG antibodies on days 0, 3, 7, and 10 of the secondary IT (n = 5). Data were collected from 4 independent experiments. (**E**) Proportion of DX5^−^ NK cells in total NK cells obtained from IT recipients together with the control or anti-TNF-α antibodies at primary and secondary IT (n = 7). Expression of CD69, TRAIL, and CXCR3 on these cells was analysed. Data are presented as the means ± standard deviation of 5 independent experiments. ^*^p < 0.05.

On the basis of above described finding that TNF-α predominantly activated DX5^−^ NK cells, anti-TNF-α antibody administration might constitute a therapeutic strategy for improving either primary or secondary islet engraftment. On the study investigating this issue, most diabetic mice that received anti-TNF-α antibodies at the both time of primary and secondary ITs became normoglycemic (86%, 6/7) (Fig. 7A), whereas none of the mice treated with control goat IgG became normoglycemic (0%, 0/7) (Fig. 7B) (p = 0.0047). Consistently, anti-TNF-α antibody administration significantly reduced the DX5^−^ NK cell proportion after secondary IT, accompanying the CD69- and TRAIL-positive NK cell proportion decreases (Fig. 7E). Anti-TNF-α antibodies on secondary IT alone did not modulate NK cell activation (Supplementary Fig. S15), and islet engraftment between recipient mice with anti-TNF-α (20%, 1/5) and control antibodies (0%, 0/5) did not significantly differ (Fig. 7C,D) (p = 1). Thus, anti-TNF-α antibody treatment may prevent NK cell activation after IT but may not calm primary IT-induced pre-activated NK cells.

## Discussion

The clinical application of IT is limited mainly owing to the early loss of transplanted islets, resulting in low transplantation efficiency. It has been demonstrated that high-mobility group box 1 (HMGB1) proteins released from transplanted islets, regardless whether allogeneic or syngeneic, trigger the activation of liver resident innate immune cells causing the early loss of transplanted islets^39^. NKT cell-dependent IFN-γ production has been proven to be essential for this process^7^. In addition, we recently showed that NK cells play a role in the early islet graft loss after syngeneic intraportal IT in an NKT cell-independent manner; *i.e*., anti-NK1.1 mAbs were shown to improve the engraftment of intraportally transplanted syngeneic islets even in NKT-deficient *CD1d*^−/−^ mice^38^.

IBMIR is a nonspecific response mediated by the innate immune system including the liver resident lymphocytes, and it is characterised by coagulation, complement activation, platelet adhesion, and leukocyte infiltration into the islets. Previous reports have demonstrated that IBMIR plays significant roles in the damaging of allogeneic, xenogeneic, and even syngeneic transplanted islets during the peritransplant period^9,40,41^. Tissue factor, expressed by the islets, is considered a major IBMIR trigger, leading to the platelet activation and release of downstream inflammatory mediators, such as TNF-α, IFN-γ, and IL-lβ^8,12^.

In this study, we showed that proinflammatory cytokines, predominantly TNF-α, further induce the activation of liver DX5^−^ NK cells that express both TNF-α 1 and 2 receptors. TNF-α has been generally regarded as a toxic cytokine mediating islet injury after IT^42,43^, and its blockade has been proven to have beneficial effects on the engraftment of transplanted islets, which led to the inclusion of this procedure in the latest IT therapy protocol Clinical Islet Transplantation 07 (CIT-07)^3,44^. Despite the clinical use, the efficacy of anti-TNF-α antibody against liver immune cells is little known with certainty. In this study, we observed that the treatment with anti-TNF-α antibody prior to IT significantly inhibits the alterations in the absolute number of DX5^−^ NK cells and CD69/TRAIL expression on liver NK cells. Therefore, we showed that TNF-α plays key roles in the activation of DX5^−^ NK cells early after IT, and that the inhibition of TNF-α has protective effects on the transplanted islets, at least partly by preventing the activation of liver NK cells during IBMIR.

Liver NK cells differ from conventional NK cells. NK cells contribute to the early defence against virus-infected and neoplastic cells based on the absent self-markers; a missing-self hypothesis had been proposed to explain the protective effects of target cell MHC class I on NK cell-mediated lysis^45,46^. We have previously demonstrated that liver resident TRAIL-positive NK cells lack the expression of Ly-49 inhibitory receptors recognising self-MHC class I, which leads to the lowering of their capacity for the self-recognition of NK cells that possess cytotoxic activity against syngeneic hepatocytes expressing DRs, which recognise TRAIL in mice^47^. We further demonstrated that NK cell-mediated cytotoxicity represents a major obstacle to the engraftment of autologous hepatocytes during hepatocyte transplantation because the direct interaction of the hepatocytes transplanted via the portal vein with liver NK cells is inevitable^47^. This self-cytotoxic mechanism may contribute to the prevention of the aberrant implantation of self-cells undesirably peeled away from the gastrointestinal tract through the portal vein. In a similar way, the transplanted islets expressing DRs also become stacked at the portal vein radices, resulting in the deposition of some cells at the hepatic sinusoid in close contact with liver TRAIL^+^ NK cells^12^. In addition to the anatomical features of sinusoids, the characteristic chemotaxis of liver DX5^−^ NK cells expressing CXCR3 contribute to the inhibition of the engraftment of transplanted islets secreting CXCL10.

Liver-resident NK cells were recently described to have the unique capacity to confer immunological memory in the form of hapten-specific contact hypersensitivity independent of T and B cells^29,48^. Peng *et al*. characterised this unique phenotype as CD49a^+^ DX5^–^ and found that they display memory response in contact hypersensitivity models^29^. They have further demonstrated that liver DX5^−^ NK cells originate primarily from the liver and are not differentiated from other NK cell subsets, including both non-liver NK cells and liver DX5^+^ NK cells. Consistent with the previous study, the adoptively transferred liver DX5^+^ NK cells did not convert into liver DX5^−^ NK cells after IT in B6 *Rag-2*^−/−^ *γ chain*^−/−^ mice. In addition, liver DX5^−^ NK cells did not show any proliferative potential after IT. This finding suggested that liver DX5^−^ NK cells are recruited from other sites after IT. We investigated whether the memory function of liver DX5^−^ NK cells activated after the primary IT affects the engraftment of secondary transplanted islets. We found that primary IT disturbed the reversal of diabetes in recipients that received a sufficient mass of secondary transplanted islets, and a significant increase in CD69, TRAIL, and CXCR3 expression was observed in liver DX5^−^ NK cells after secondary IT, in comparison with that in the control group that did not receive the primary IT, indicating their memory-like function. There may be a possibility that the inflammation owing to the primary IT remains exist at the time of secondary IT or that the other immune cell memory function is involved in secondary islet rejection. To investigate the direct evidence for the role of memory DX5^−^ NK cells in secondary islet rejection, we designed an experimental model to evaluate islet engraftment in the memory-like DX5^−^ NK cell-transferred new host. However, the difference in chemotactic responses between DX5^−^ NK and DX5^+^ NK cells and the spontaneous activation of NK cells in consequence of adoptive transfer did not allowed us to compare the islet engraftment in the liver between DX5^−^ NK- and DX5^+^ NK cell-transferred hosts. Hence, we assessed the role of memory NK cells in secondary islet rejection by depleting NK cells prior to secondary IT. NK cell depletion improved the engraftment of 400 secondary transplanted islets, suggesting that liver DX5^−^ NK cell activation was accelerated as a recall response after secondary IT. Considering the characteristic deployment of memory-like DX5^−^ NK cells in the liver, this organ may not be the optimal site for islet infusion. Alternative implantation sites, such as subcutaneous tissue, omentum, and intramuscular tissue, have been investigated^13,14,49^. The effects of innate immune response on islets transplanted into these anatomical sites should be further investigated.

In this study, it is notable that the liver DX5^−^ NK cell activation during IBMIR led to long-lasting NK cell cytotoxic activity and amplified their activation against secondary transplanted islets as a recall response even after syngeneic IT. Liver DX5^−^ NK cell activation after allogeneic IT was comparable to that in syngeneic IT, indicating that an NK cell innate immunity-induced barrier constitutes a common feature of both types of IT. However, based on the possible collaboration between innate and acquired immunity, NK cell activation might accelerate subsequent alloimmune responses. Further investigations are required to clarify this issue. The regulation of the activity of these memory-like NK cells by anti-TNF-α treatment may improve the rates of engraftment of sequentially transplanted islets. However, the selective depletion of liver DX5^−^ NK cells remains difficult in clinical settings. Thus, preventing direct interaction between liver NK cells and transplanted islets through the inhibition of TRAIL-DR and/or CXCR3-CXCL10 pathways may represent a promising approach.

## Methods

### Mice

We purchased 8–12-week-old male C57BL/6J (B6) and BALB/c mice from Clea Japan, Inc. (Osaka, Japan), B6 *Rag-2*^−/−^ *γ chain*^−/−^ mice from Taconic Farms (Hudson, NY, USA), and B6 *CXCR3*^−/−^ and *Tbx21*^−/−^ mice from The Jackson Laboratory (Bar Harbor, ME, USA). All mice were housed in the animal facility of Hiroshima University, Japan, in a pathogen-free, micro-isolated environment. This study and all experiments were approved by the institutional review board of Hiroshima University and conducted according to the guidelines of the National Institutes of Health (publication no. 86–23, revised 1996).

### Antibodies and flow cytometry analysis

Flow cytometry was performed with a FACS Canto II and FACS Aria II (BD Biosciences, Mountain View, CA, USA), and the obtained results were analysed using FlowJo software (Tree Star Software, San Carlos, CA, USA). LMNCs were stained using the following monoclonal antibodies (mAbs): fluorescein isothiocyanate (FITC)-conjugated anti-DX5, APC-conjugated anti-NK1.1, APC-conjugated anti-CD120a, APC-Cy7-conjugated anti-T cell receptor (TCR)-β, PE-conjugated anti-CD69, PE-conjugated anti-CXCR3, PE-conjugated anti-natural killer group 2, member D (NKG2D), PE-conjugated anti-NK1.1, PE-conjugated anti-CD120b (all from BD Pharmingen, San Diego, CA, USA), and PE-conjugated anti-TRAIL (BioLegend, San Diego, CA, USA). Proliferative potential was tested by intracellular staining using FITC-conjugated anti- Ki-67 (Miltenyi Biotec, Auburn, CA, USA) or isotype control. Nonspecific FcγR binding of labelled mAbs was blocked by anti-CD16/32 (2.4G2) (BD Pharmingen). Dead cells were excluded from the analysis by light-scatter and/or propidium iodide staining.

### Liver NK cell isolation

LMNCs were prepared as previously described^50^. Briefly, the liver was removed after perfusion with 1 mL phosphate-buffered saline (PBS) supplemented with 10% heparin via the portal vein. LMNCs were obtained from the liver by perfusion with 50 mL PBS supplemented with 0.1% ethylenediaminetetraacetic acid (EDTA; Sigma-Aldrich, St. Louis, MO, USA). For *in vitro* culturing, LMNCs were separated into NK and non-NK cell fractions using the Mouse NK Cell Isolation Kit II (Miltenyi Biotec). Only the samples with more than 90% purity of isolated fractions were used for further analyses.

### Adoptive transfer

LMNCs from B6 *wild-type* mice were stained with APC-conjugated anti-mouse NK1.1, APC-Cy7-conjugated anti-TCRβ, and FITC-conjugated anti-DX5 antibodies. Afterward, liver DX5^+^ or DX5^−^ NK cells were isolated by FACS Aria II sorting. The isolated cells (0.2 × 10^6^) were intravenously injected into B6 *Rag-2*^−/−^ *γ chain*^−/−^ mice.

### Cell culture

Isolated liver NK cells were cultured with the following cytokines: tumour necrosis factor-α (TNF-α) (50 ng/mL), interferon-γ (IFN-γ) (1 µg/mL), and IL-lβ (25 ng/mL; all from R&D Systems, Minneapolis, MN, USA), in RPMI 1640 medium supplemented with 10% heat-inactivated fetal calf serum (Sanko Chemical Co., Ltd., Tokyo, Japan), 25 mmol/L HEPES Buffer (Gibco, Grand Island, NY, USA), 50 mol/L 2-mercaptoethanol (Katayama Chemical Co., Osaka, Japan), 50 U/mL penicillin, and 50 g/mL streptomycin (Gibco) (hereafter referred to as cRPMI) at 37 °C in a 5% CO_2_ incubator. After 24 h incubation, the cultured cells were harvested for further analyses.

### Islet isolation and transplantation

Islets were isolated using our standard procedure^6^. Islets 100- to 200-µm in diameter were suspended in 200 µL Hank’s balanced salt solution (Gibco) and transplanted into the liver via the portal vein^51^. Streptozotocin (STZ, 200 mg/kg; Sigma-Aldrich) was intraperitoneally administered to recipient mice to induce diabetes 7 days before IT. Blood glucose levels were measured using a GT-1830 glucose analyser (Arkray, Tokyo, Japan). Non-fasting blood glucose levels exceeded 500 mg/dL by day 3 after STZ injection and mice remained hyperglycaemic until IT. Blood glucose levels shown to decrease below 200 mg/dL by two consecutive measurements indicated the reversal of diabetes mellitus. We previously showed that transplanting 400 syngeneic islets into the liver was sufficient to reverse hyperglycaemia in diabetic recipient mice^38^ whereas transplanting 300 or 200 syngeneic islets was considered as a marginal or insufficient mass of islets to achieve normoglycemia in diabetic recipient mice, respectively.

### Luminescent cytotoxicity assay for islets

NK cell cytotoxicity against islets was measured using the CytoTox-Glo Cytotoxicity Assay (Promega, Madison, WI, USA), which measures cell death through the release of dead-cell protease activity from dying cells that have lost membrane integrity. As previously described^6^, 0.2 × 10^6^ isolated NK cells were used as effector cells and two islets served as target cells. After 8-h incubation, data was collected using the GloMax Discover System (Promega). In some experiments, the assay was performed in the presence of 10 µg/mL of anti-TRAIL mAb (BD Pharmingen), 2 µg/mL of anti-IFN-γ mAb (R&D), and 50 nmol/L of concanamycin A (Sigma–Aldrich). The average culture medium background luminescence value was subtracted from experimental (A), target cell spontaneous (B), effector cell spontaneous (C), and target cell maximum (D) release. Cytotoxicity percentage was calculated as % cytotoxicity = (A − B − C)/(D − B) × 100.

### NK cell depletion

For NK cell-depleting experiments, B6 mice were treated with intraperitoneal injection of rabbit anti-asialo GM1 serum (50 µL/mouse; Wako Pure Chemicals Industries, Richmond, VA, USA) on days −3, 0, and 14 of secondary IT.

### TNF-α neutralising antibody treatment

Specific neutralising antibodies against TNF-α (100 µg per mouse; R&D Systems) were administered by systemic intraperitoneal injection into B6 mice 24 h before IT. For secondary IT experiments, TNF-α neutralising antibodies were administered on days 0, 3, 7, and 10 of IT. Control mice were injected with normal goat IgG (R&D Systems).

### Real-Time RT-PCR

Real-time RT-PCR was performed for TNF-α, IFN-γ, IL-1β, and beta-2-microglobulin as the housekeeping gene. Liver samples were harvested after IT and stored until use. Total mRNA was extracted using an RNeasy Mini kit (Qiagen, Valencia, CA, USA) according to the manufacturer’s protocol. cDNA was generated using a QuantiTect Reverse Transcription Kit (Qiagen) and amplified with a Rotor-Gene Q 2PLEX HRM Real-Time PCR system (Qiagen). TNF-α, IFN-γ, and IL-1β expressions were investigated using appropriate primers and probes (Taqman Universal PCR MasterMix, TaqmanGene Expression Assays, Applied Biosystems) with 32 ng of reverse transcribed total RNA in a total volume of 25 µl (Supplementary Table S1). The amplification protocol consisted of denaturation at 95 °C for 5 min, followed by 40 cycles of 95 °C for 5 s and 60 °C for 10 s.

### Statistical analysis

Data were presented as the means ± standard deviation (SD). Continuous and dichotomous variables were compared using unpaired Student’s *t*-tests and Fisher’s exact tests, respectively. For three or more group comparisons, statistical significance was determined using one-way ANOVA with Tukey post hoc analysis. All statistical analyses were performed using statistical software JMP version 10 (SAS Institute, Cary, NC, USA). A p-value of < 0.05 was considered statistically significant.

## Supplementary information

Supplementary Information
