# Supplementary material for: Memory-like Liver Natural Killer Cells are Responsible for Islet Destruction in Secondary Islet Transplantation
Source: Sci Rep. 2019 Jan 31;9:1022. doi: 10.1038/s41598-018-37395-9 (PMC6355863; doi:10.1038/s41598-018-37395-9)
Supplement: Supplementary file 1 — Supplementary Information [file 41598_2018_37395_MOESM1_ESM.pdf]

Memory-like Liver Natural Killer Cells are Responsible for Islet Destruction in Secondary Islet Transplantation

Y. Saeki, K. Ishiyama, N. Ishida, Y. Tanaka, and H. Ohdan

Supplementary Material and Methods

Migration assays

To assess migration, 15 islets were plated in the outer well of a 24-well transwell plate (Costar, Corning, NY, USA) in a final volume of 600 μL cRPMI, before being incubated at 37°C and 5% CO 2 for 1 h. Isolated NK cells (0.7×10^6 cells) were then added in a volume of 100 μL to the 5-μm–pore transwell insert. The number of NK cells following a 2-h incubation was counted by light microscopy. The number of transmigrated cells was calculated by subtracting the number of remaining cells in the transwell insert and NK cells that migrated in response to medium alone from the number of seeded cells.

Cytometric beads array for cytokine measurements

Cytokine serum levels were determined using mouse Cytometric Beads Array (CBA) Flex set (BD Biosciences Pharmingen, USA) to quantify TNF-α, IFN-γ, and IL-lβ according to the manufacturer instructions.

NK cell depletion

To deplete NK cells, B6 mice were treated with intraperitoneal injection of rabbit anti-asialo GM1 serum (50 μL/mouse; Wako Pure Chemicals Industries, Richmond, VA, USA) or that of anti-NK1.1 mAb (300 μg per mouse) 2 days before analysis. Anti-NK1.1 mAb was prepared in the laboratory from a hybridoma (PK136; ATCC).

Supplementary Table S1. Primer and probe sequences for the cytokines and β-2 microglobulin

Supplementary Figure S1: $DX5^-$ and $DX5^+$ subsets of natural killer (NK) cells. (**A**) The proportion of $TCR\beta^-$ $NK1.1^+$ $DX5^-$ NK cells among total liver and splenic NK cells obtained from naive C57BL/6 (B6) mice is shown as the means ± standard deviation (SD) of 6 independent experiments (n = 7). $^*p < 0.001$. (**B**) Phenotypic difference between liver $DX5^-$ NK cells and $DX5^+$ NK cells. Representative data of 6 independent experiments are included (n = 6–7). Solid lines, staining of the indicated molecules on $DX5^-$ NK cells; dashed lines, staining of the indicated molecules on $DX5^+$ NK cells. Gray-shaded histograms represent isotype controls. Mean fluorescence intensity with SD of indicated molecules on each NK cell subset is provided. (**C**) The proportions of the NK cells positive for the indicated molecules are shown in bar graphs ($DX5^-$ NK cells, open bar; $DX5^+$ NK cells, solid bar) as the means ± SD of 6 independent experiments (n = 6–7). $^*p < 0.001$.

Supplementary Figure S2: Liver NK cell chemotactic responses to islets. (**A**) Proportion of TCRβ$^-$ NK1.1$^+$ CXCR3$^+$ NK cells among total liver NK cells obtained from naive C57BL/6 (B6) *wild-type* and B6 *CXCR3*$^{-/-}$ mice. Gray-shaded histograms represent staining of the CXCR3 on liver NK cells. Dashed lines represent isotype controls. (**B**) Number of transmigrated cells are shown in bar graphs as the mean ± standard deviation of 2 independent experiments (n = 3). $^*p < 0.05$.

Supplementary Figure S3: Phenotypic alterations of liver natural killer (NK) cells after adoptive transfer. TCRβ$^-$ NK1.1$^+$ NK cells were isolated from liver mononuclear cells (LMNCs) of C57BL/6 (B6) *wild-type* mice and were transferred into B6 *Rag-2*$^{-/-}$ *γ chain*$^{-/-}$ mice, and LMNCs from the recipient mice were analysed by using flow cytometry after 14 days. (**A**) Representative dot plots show the isolated NK cells and transferred NK cells. Bar graph representing the mean percentage ± standard deviation (SD) of the proportion of DX5$^-$ NK cells among total liver NK cells before or after adoptive transfer (n = 6). Data were collected from 2 independent experiments. ** $p < 0.000001$. (**B**) Percentages of CD69-, TRAIL-, or CXCR3-positive NK cells in the liver before and after adoptive transfer were analysed using flow cytometry (isolated NK cells, open bar; transferred NK cells, solid bar) (n = 6). The data in bar graphs are shown as the means ± SD of 2 independent experiments. $^*p < 0.01$. $^{**}p < 0.000001$. (**C**) Phenotypic difference of liver DX5$^-$ NK cells and DX5$^+$ NK cells between before and after adoptive transfer. Representative data of 2 independent experiments are included (n = 6). Solid lines, staining of the indicated molecules on DX5$^-$ NK cells; dashed lines, staining of the indicated molecules on DX5$^+$ NK cells. Gray-shaded histograms represent isotype controls. Mean fluorescence intensity with SD of indicated molecules on each NK cell subset is provided.

Supplementary Figure S4: Serum levels of the TNF-α, IFN-γ, and IL-1β during instant blood-mediated inflammatory reaction (IBMIR). Serum was harvested from C57BL/6J mice that received 300 syngeneic islets 6, 12, 24, 48, and 72 h after islet transplantation. Serum levels of pro-inflammatory (TNF-α, IFN-γ, and IL-lβ) cytokines were determined by cytometric beads array. The data in bar graphs are presented as the means ± standard deviation (n = 3–4).

Supplementary Figure S5: TNF-α receptor (TNFR) expression on liver $DX5^{-}$ NK cells. Proportion of $TCR\beta^{-}$ $NK1.1^{+}$ $TNFR^{+}$ NK cells among total liver $DX5^{-}$ cells obtained from naive C57BL/6 mouse. Gray-shaded histograms represent staining of the TNFR1 or TNFR2 on liver $DX5^{-}$ NK cells. Dashed lines represent isotype controls.

Supplementary Figure S6: Phenotypic alterations of liver $DX5^{-}$ and $DX5^{+}$ natural killer (NK) cells during instant blood-mediated inflammatory reaction. Liver $TCR\beta^{-}$ $NK1.1^{+}$ NK cells were separated from liver mononuclear cells and cultured in the presence of indicated cytokines among TNF-α, IFN-γ, and IL-1β. Cells were harvested after 24 h and analysed with flow cytometry. (**A**) Survival ratio of seeded NK cells was obtained by dividing the number of surviving NK cells by the number of seeded NK cells. The data in bar graphs are presented as the means ± standard deviation (SD) of 4 independent experiments (n = 4−5). (**B**) Survival ratio of seeded $DX5^{-}$ and $DX5^{+}$ NK cells. The data in bar graphs ($DX5^{-}$ NK cells, solid bar; $DX5^{+}$ NK cells, open bar) are presented as the means ± SD of 4 independent experiments (n = 4–5). (**C** and **D**) Liver NK cells treated with the cytokine combinations for 24 h (n = 4–5). The data in bar

graphs are presented as the means ± SD of 4 independent experiments. $^{*}p < 0.05$; $^{**}p < 0.01$. $^{\#}p < 0.05$; $^{\#\#}p < 0.01$; $^{\#\#\#}p < 0.001$, compared with the results of liver NK cells without cytokines.

Supplementary Figure S7: Phenotypic alteration of DX5 on liver natural killer (NK) cells early after islet transplantation (IT). A C57BL/6 (B6) *wild-type* mouse was intraportally transplanted with 300 syngeneic islets, and harvested 24 h after IT. Phenotypic alterations of DX5 on TCRβ^{-} NK1.1^{+} NK cells in the livers from B6 mice with or without intraportal IT were analysed with flow cytometry. Representative data of 5 independent experiments are included (n = 5). Gray-shaded histograms represent staining of DX5 on liver NK cells. Dashed lines represent isotype controls. Mean fluorescence intensity with SD of indicated molecules on each NK cell subset is provided.

Supplementary Figure S8: Phenotypic alterations of liver DX5^{-} and DX5^{+} natural killer (NK) cells early after islet transplantation (IT). C57BL/6 *wild-type* mice administered PBS or anti-TNF-α antibody were treated with 300 syngeneic islets. Phenotypic alterations of DX5^{-} and DX5^{+} TCRβ^{-} NK1.1^{+} NK cells in the liver were analysed 24 h after intraportal IT. (**A**, **C**, **E**, and **G**) Percentages of CD69-, TRAIL-, CXCR3-, or NKG2D positive DX5^{-} NK cells in the liver after intraportal IT were analysed with flow cytometry (naive group, open bar; group that received transplantation, solid bar; group that received islet and anti-TNF-α antibody treatment, gray bar) (n = 5–7). The data in bar graphs are shown as the means ± standard deviation (SD) of 5 independent experiments. $^{*}p < 0.05$. $^{**}p < 0.001$. (**B**, **D**, **F**, and **H**) Percentages of CD69-, TRAIL-, CXCR3-, or NKG2D positive DX5^{+} NK cells in the liver after intraportal IT were

analysed with flow cytometry (naive group, open bar; group that received transplantation, solid bar; group that received islet and anti-TNF-α antibody treatment, gray bar) (n = 5–7). The data in bar graphs are shown as the means ± SD of 5 independent experiments.

Supplementary Figure S9: Phenotypic alterations of liver $DX5^{-}$ and $DX5^{+}$ natural killer (NK) cells early after islet transplantation (IT). C57BL/6 *wild-type* mice administered PBS or anti-TNF-α antibody were treated with 300 syngeneic islets. Phenotypic alterations of $DX5^{-}$ and $DX5^{+}$ $TCR\beta^{-}$ $NK1.1^{+}$ NK cells in the liver were analysed 24 h after intraportal IT. (**A**) Representative dot plots of NK1.1 and DX5 in $TCR\beta^{-}$ $NK1.1^{+}$ NK cells in the liver after intraportal IT. (**B**) Phenotypic difference of liver $DX5^{-}$ NK cells and $DX5^{+}$ NK cells between before and after adoptive transfer. Representative data of 5 independent experiments are included (n = 5-7). Solid lines, staining of the indicated molecules on $DX5^{-}$ NK cells; dashed lines, staining of the indicated molecules on $DX5^{+}$ NK cells. Gray-shaded histograms represent isotype controls. Mean fluorescence intensity with SD of indicated molecules on each NK cell subset is provided.

Supplementary Figure S10: Immune state after allogeneic islet transplantation (IT). C57BL/6 (B6) *wild-type* mice were transplanted with 300 islets from B6 (syngeneic) or BALB/c (allogeneic) mice. Phenotypic alterations of immune cells in the liver were analysed with flow cytometry 24 h after intraportal IT (syngeneic group, open bar; allogeneic group, solid bar) (n = 5). Data were collected from 3 independent experiments (n = 5). (**A**) Proportion of $TCR\beta^{-}$ $NK1.1^{+}$ NK cells, $TCR\beta^{+}$ $NK1.1^{+}$ NKT cells, and $TCR\beta^{+}$ $NK1.1^{-}$ T cells in total lymphocytes

obtained from the livers of mice that received 300 syngeneic or allogeneic islets. The data in bar graphs are shown as the means ± standard deviation (SD). (**B** and **D**) Absolute number of NK cells and $DX5^{-}$ NK cells obtained from the livers of mice that received 300 syngeneic or allogeneic islets. The data in bar graphs are shown as the means ± SD. (**C**) Proportion of $DX5^{-}$ NK cells in total NK cells obtained from the livers of mice that received 300 syngeneic or allogeneic islets. The data in bar graphs are shown as the means ± SD. (**E**, **F**, and **G**) Percentages of CD69-, TRAIL-, and CXCR3 positive NK cells obtained from the livers of mice that received 300 syngeneic or allogeneic islets. The data in bar graphs are shown as the means ± SD.

Supplementary Figure S11: Number of transferred liver $DX5^{-}$ NK cells in the new host that received islet transplantation (IT). $TCR\beta^{-}$ $NK1.1^{+}$ $DX5^{-}$ NK cells were isolated from liver mononuclear cells (LMNCs) of C57BL/6 (B6) *wild-type* mice. Isolated $DX5^{-}$ NK cells were transferred into B6 *Rag-2*$^{-/-}$ *γ chain*$^{-/-}$ mice, which was followed by IT, after which LMNCs from the recipient mice were analysed. The absolute number of $DX5^{-}$ NK cell obtained from the whole liver of $DX5^{-}$ NK cell-transferred recipients with/without IT is shown. The data in bar graphs are shown as the means ± standard deviation of 2 independent experiments (n = 5).

Supplementary Figure S12: Proliferative potential of liver natural killer (NK) cells after islet transplantation (IT). (**A**, **B**, and **C**) C57BL/6 *wild-type* mice were treated with 300 syngeneic islets. $TCR\beta^{-}$ $NK1.1^{+}$ NK cells were gated on $DX5^{-}$ and $DX5^{+}$ NK cells. Ki-67 expression on total NK cells and in each subset was assessed. Percentages of Ki-67 positive NK cells in the liver after intraportal IT were analysed with flow cytometry (naive group, open bar;

group that received transplantation, solid bar) (n = 6). The data in bar graphs are shown as the means ± standard deviation (SD) of 3 independent experiments.

Supplementary Figure S13: Phenotypic alterations of liver DX5^{-} and DX5^{+} natural killer (NK) cells at the late phase after islet transplantation (IT). Phenotypic alterations of DX5^{-} and DX5^{+} TCRβ^{-} NK1.1^{+} NK cells in the liver from diabetic C57BL/6 (B6) mice were analysed 14 days (n = 6, 3 independent experiments) or 35 days (n = 8, 6 independent experiments) after intraportal IT of syngeneic 300 islets. The data from transplanted diabetic mice are compared with the data from control diabetic B6 mice. Diabetic B6 mice treated with streptozotocin 7 days before were used as recipients. (**A**, **C**, and **E**) Proportion of liver DX5^{-} NK cells positive for markers at the indicated time points. $^{*}p < 0.05$; $^{**}p < 0.001$, compared with control. (**B**, **D**, and **F**) Proportion of liver DX5^{+} NK cells positive for markers at the indicated time points.

Supplementary Figure S14: The proportion of lymphocytes in anti-ASGM1 antibody or anti-NK1.1 antibody-treated wild-type mice. Mononuclear cells (MNCs) were collected from wild-type, anti-ASGM1 antibody, or anti-NK1.1 antibody-treated wild-type mice 2 days after treatment. (A) Representative dot plots of NK cells and NKT cells in liver MNCs. MNCs were stained for NK1.1, T-cell receptor (TCR) β, and **α**-GalCer-CD1d tetramer. TCRβ^{-} NK1.1^{+} NK cells and TCRβ^{+} **α**-GalCer-CD1d tetramer^{+} NKT cells from the liver were sorted with the use of flow cytometry (n = 5). (B) The proportion of TCRβ^{-} NK1.1^{+} NK cells and TCRβ^{+} **α**-GalCer-CD1d tetramer^{+} NKT cells among total liver MNCs obtained from naive, anti-ASGM1 antibody, or anti-NK1.1 antibody-treated wild-type C57BL/6 (B6) mice is shown in bar graphs as the

means ± standard deviation (naive mice, open bar; anti-ASGM1 antibody-treated mice, solid bar; anti-NK1.1 antibody-treated mice, gray bar) ($n = 5$). Data were collected from 2 independent experiments. $^{*}p < 0.001$; $^{**}p < 0.00001$; $^{***}p < 0.000001$, compared with naive.

Supplementary Figure S15: Phenotypic alterations of liver natural killer (NK) cells after secondary islet transplantation (IT) with anti-TNF-α antibodies. Diabetic C57BL/6 (B6) mice were intraportally transplanted with 400 syngeneic islets 14 days after the primary transplantation of 200 syngeneic islets. Liver TCRβ^{-} NK1.1^{+} NK cells were harvested at day 42 and analysed with flow cytometry. Diabetic B6 mice were treated with intraperitoneal injection of normal goat IgG or anti-TNF-α antibodies on days 0, 3, 7, and 10 of the secondary IT (group that received islets and control antibodies, open bar; group that received islets and anti-TNF-α antibodies, solid bar) ($n = 5$). Data were collected from 4 independent experiments. (**A**) Absolute number of NK cells obtained from the livers of recipient mice. The data in bar graphs are shown as the means ± standard deviation (SD). (**B** and **C**) Proportion of DX5^{-} NK cells in total NK cells and the absolute number of DX5^{-} NK cells obtained from the liver of recipient mice. The data in bar graphs are shown as the means ± SD. (**D**, **E**, and **F**) Percentages of CD69-, TRAIL-, or CXCR3-positive NK cells in the liver of recipient mice were analysed with flow cytometry. The data in bar graphs are shown as the means ± SD.

Table S1. Primer and probe sequences for the cytokines and β-2 microglobulin

Gene symbol	Gene name		Primers (5’-3’)
Tnfa	tumour necrosis factor a	F	CATCTTCTCAAAATTCGAGTGACAA
		R	TGGGAGTAGACAAGGTACAACCC
Ifng	Interferon gamma	F	TCAAGTGGCATAGATGTGGAAGAA
		R	TGGCTCTGCAGGATTTTCATG
Il1b	interleukin 1 beta	F	CGTTCCCATTAGACAACTGCACTA
		R	TGTTGGTTGATATTCTGTCCATTGA
B2m	beta-2 microglobulin	F	TGGTCTTTCTGGTGCTTGTC
		R	GTATGTTCGGCTTCCCATTC

F, Forward; R, Reverse

Figure S1

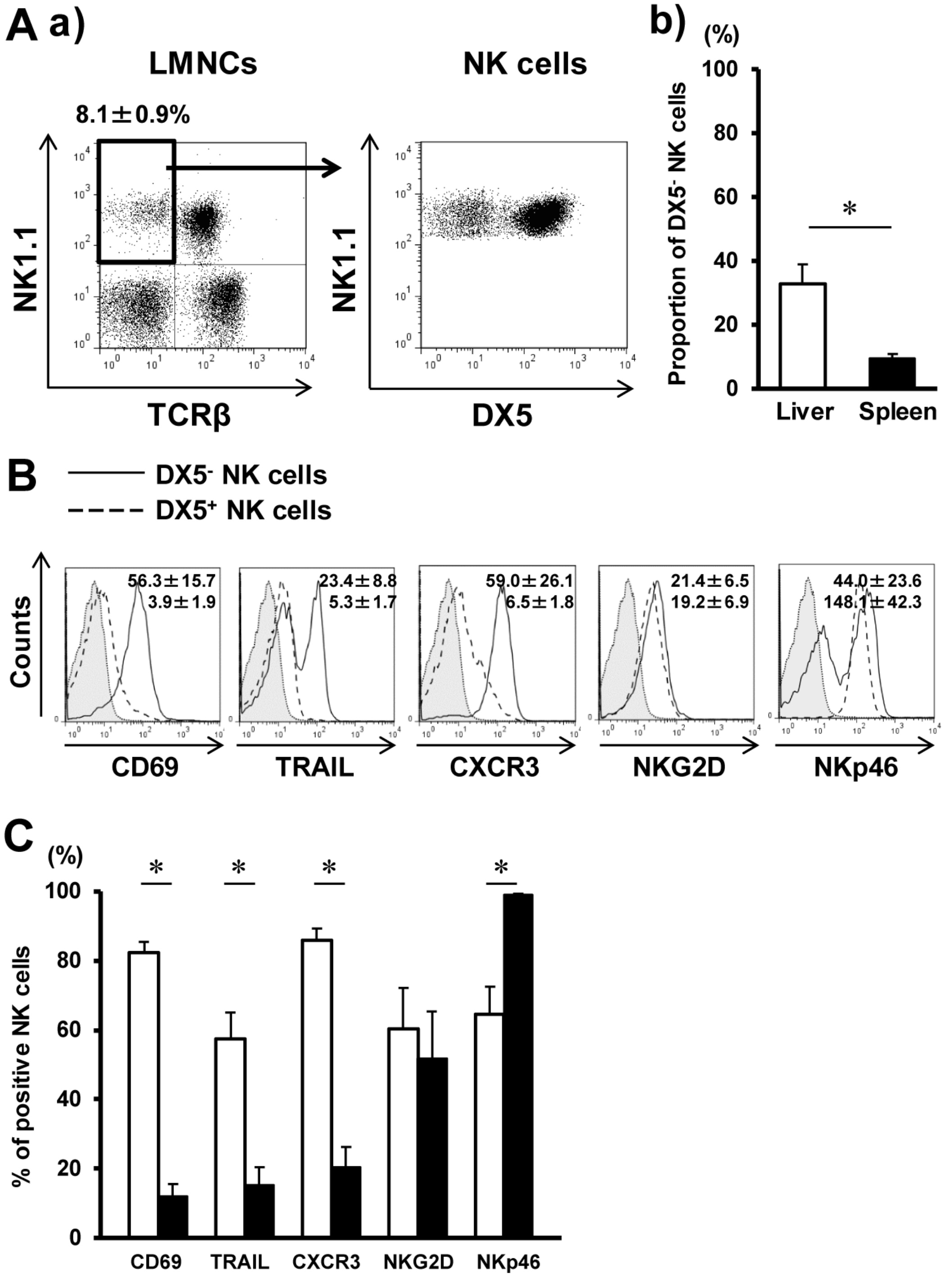
A a)
LMNCs
NK cells
8.1±0.9%
NK1.1
TCRβ
NK1.1
DX5
b)
(%)
Proportion of DX5⁻ NK cells
100
80
60
40
20
0
*
Liver
Spleen
B
DX5⁻ NK cells
DX5⁺ NK cells
Counts
56.3±15.7
3.9±1.9
23.4±8.8
5.3±1.7
59.0±26.1
6.5±1.8
21.4±6.5
19.2±6.9
44.0±23.6
148.1±42.3
CD69
TRAIL
CXCR3
NKG2D
NKp46
C
(%)
% of positive NK cells
100
80
60
40
20
0
*
*
*
*
CD69
TRAIL
CXCR3
NKG2D
NKp46

Figure S2

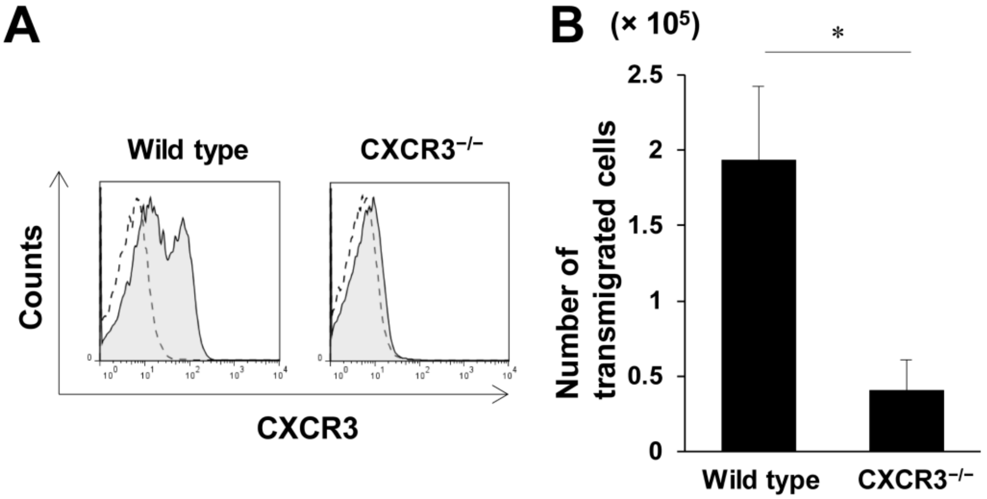
A
Wild type
CXCR3−/−
Counts
CXCR3
B
(× 10⁵)
*
2.5
2
1.5
1
0.5
0
Number of transmigrated cells
Wild type
CXCR3−/−

Figure S3

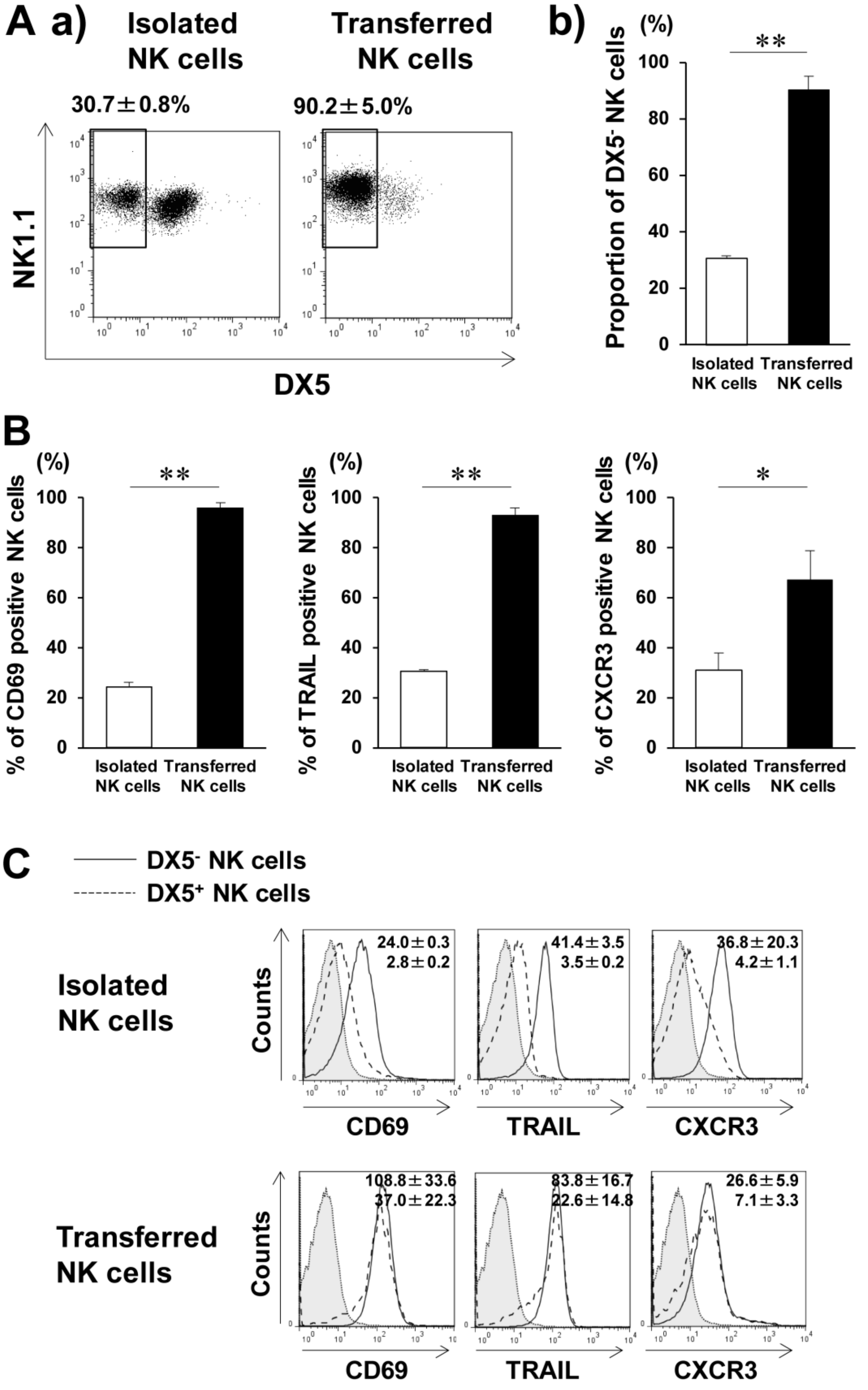

A a)
Isolated NK cells
Transferred NK cells
30.7±0.8%
90.2±5.0%
NK1.1
DX5
b)
(%)
Proportion of DX5⁻ NK cells
Isolated NK cells
Transferred NK cells
B
% of CD69 positive NK cells
% of TRAIL positive NK cells
% of CXCR3 positive NK cells
C
DX5⁻ NK cells
DX5⁺ NK cells
Isolated NK cells
Transferred NK cells
Counts
CD69
TRAIL
CXCR3
24.0±0.3
2.8±0.2
41.4±3.5
3.5±0.2
36.8±20.3
4.2±1.1
108.8±33.6
37.0±22.3
83.8±16.7
22.6±14.8
26.6±5.9
7.1±3.3

Figure S4

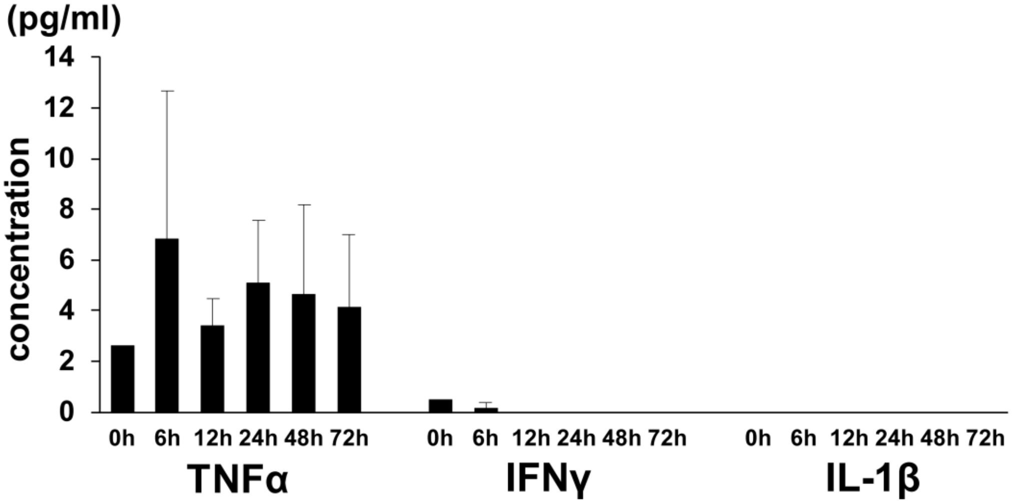
(pg/ml)
concentration
14
12
10
8
6
4
2
0
0h 6h 12h 24h 48h 72h
TNFα
0h 6h 12h 24h 48h 72h
IFNγ
0h 6h 12h 24h 48h 72h
IL-1β

Figure S5

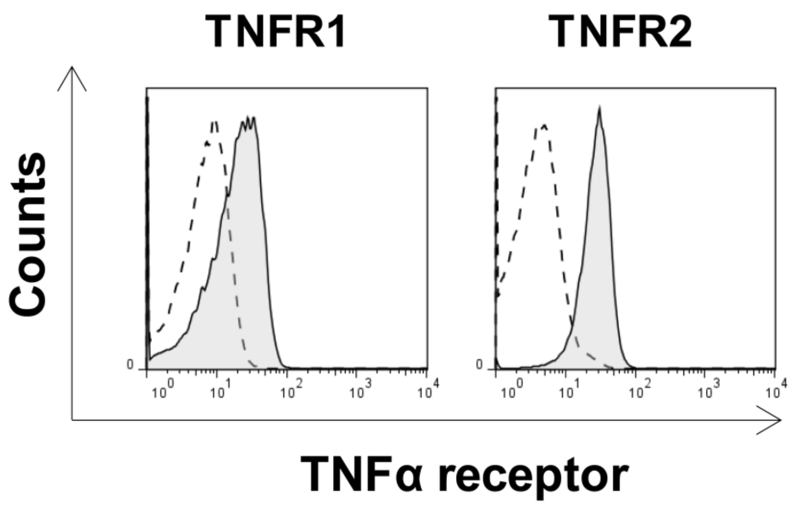
TNFR1
TNFR2
Counts
0
10^0 10^1 10^2 10^3 10^4
0
10^0 10^1 10^2 10^3 10^4
TNFα receptor

Figure S6

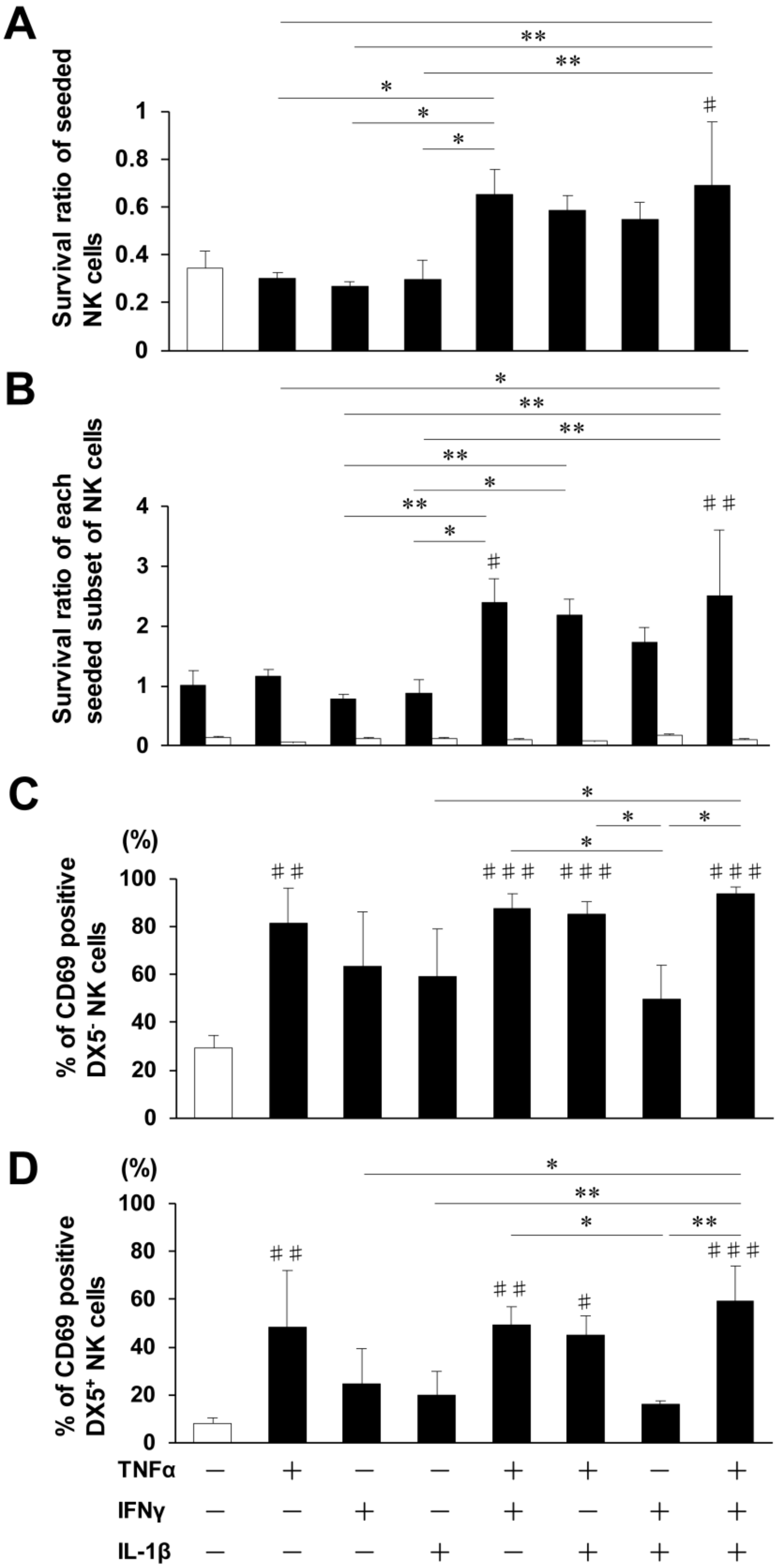
A
B
C
D
Survival ratio of seeded NK cells
Survival ratio of each seeded subset of NK cells
% of CD69 positive DX5⁻ NK cells
% of CD69 positive DX5⁺ NK cells
(%)
TNFα
IFNγ
IL-1β

Figure S7

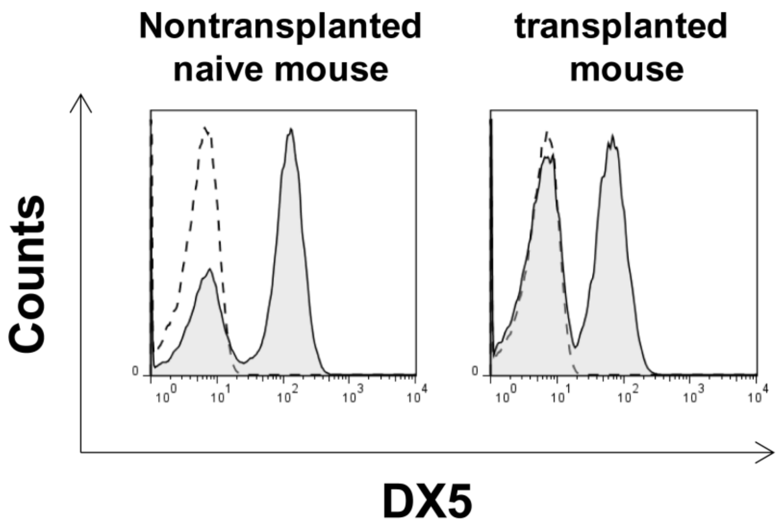

Nontransplanted naive mouse
transplanted mouse
Counts
DX5

Figure S8

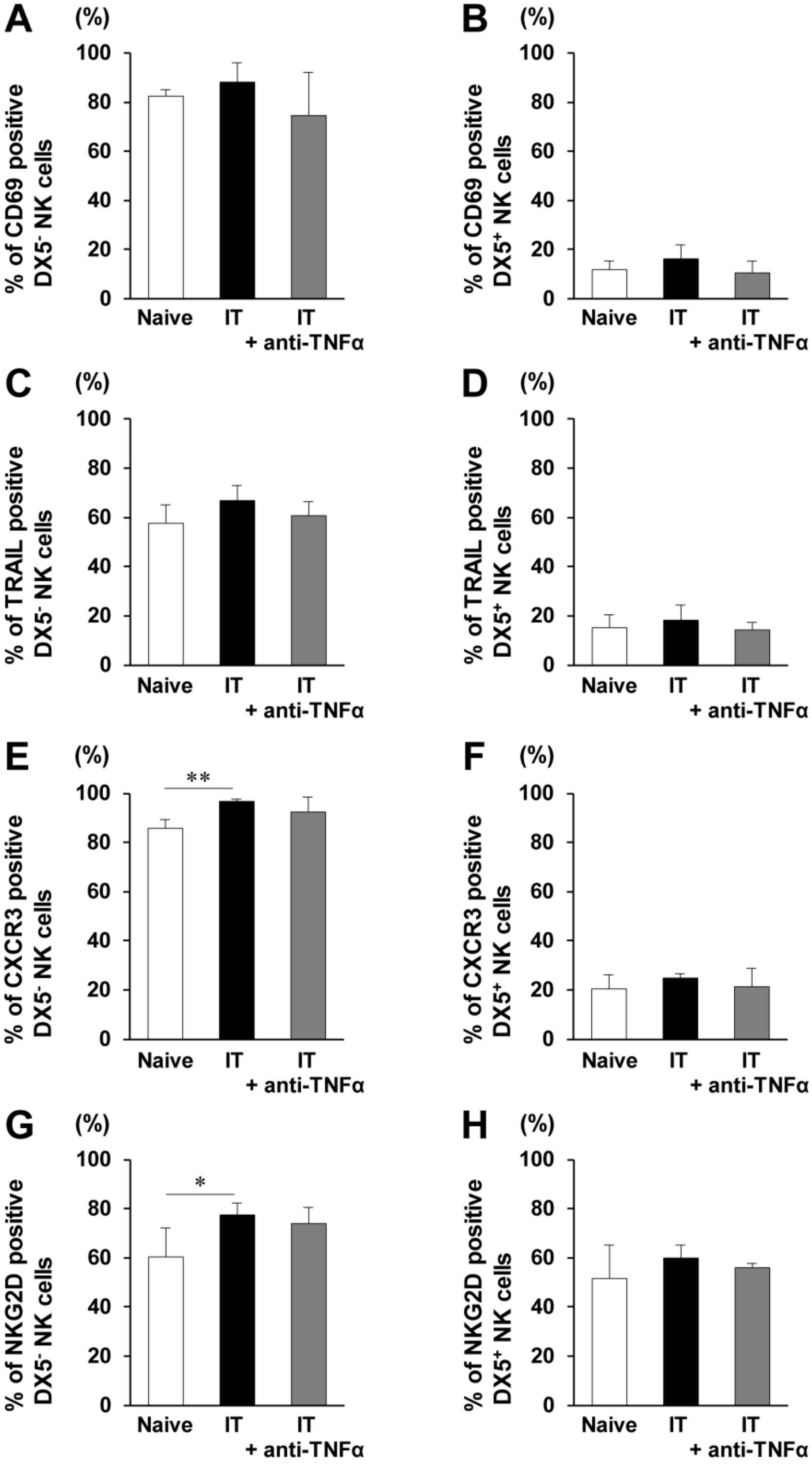

A
(%)
100
80
60
40
20
0
% of CD69 positive DX5- NK cells
Naive
IT
IT + anti-TNFα
B
(%)
% of CD69 positive DX5+ NK cells
C
(%)
% of TRAIL positive DX5- NK cells
D
(%)
% of TRAIL positive DX5+ NK cells
E
(%)
**
% of CXCR3 positive DX5- NK cells
F
(%)
% of CXCR3 positive DX5+ NK cells
G
(%)
*
% of NKG2D positive DX5- NK cells
H
(%)
% of NKG2D positive DX5+ NK cells

Figure S9

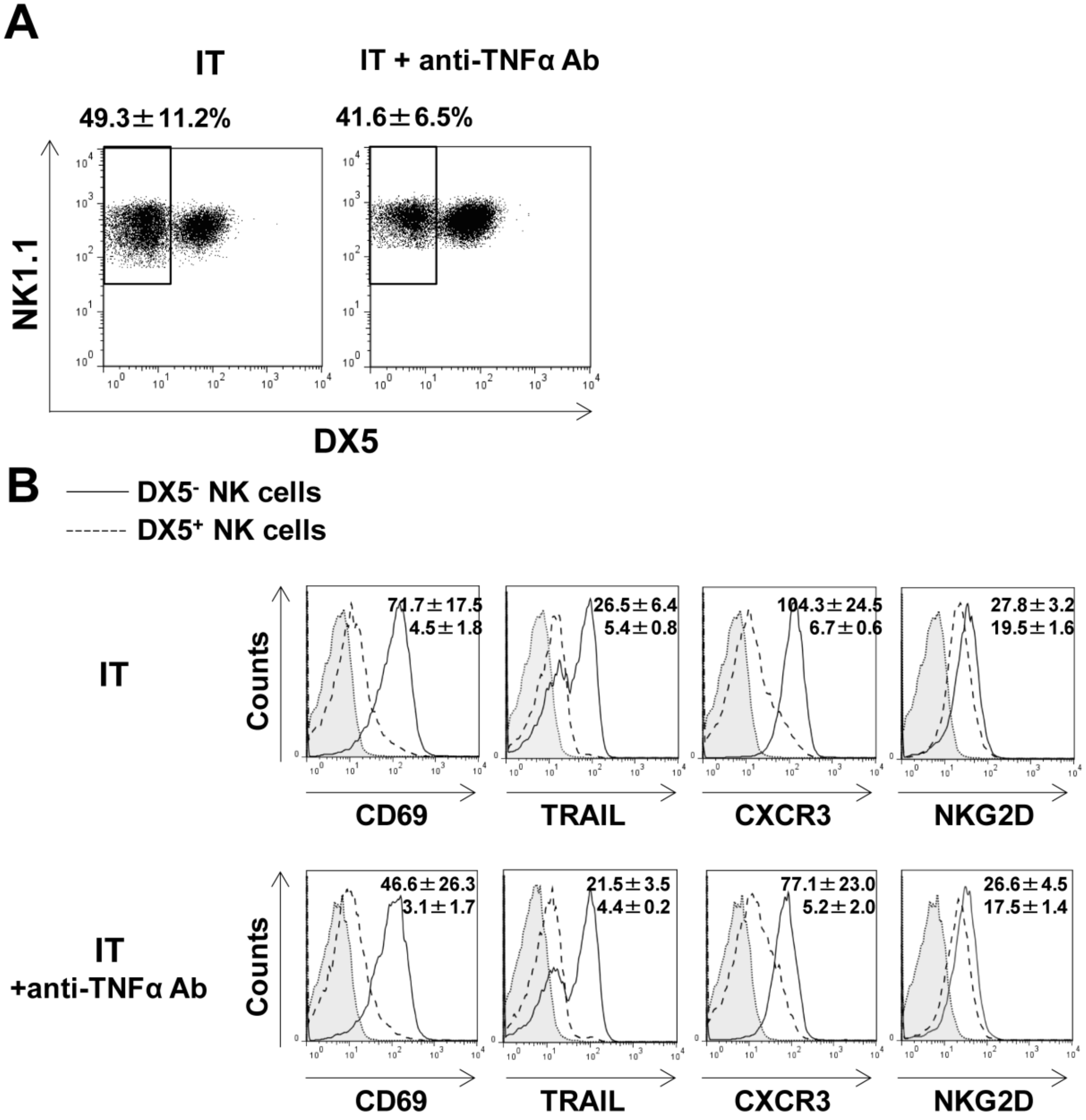
A
IT
IT + anti-TNFα Ab
49.3±11.2%
41.6±6.5%
NK1.1
DX5
B
DX5⁻ NK cells
DX5⁺ NK cells
IT
IT
+anti-TNFα Ab
Counts
CD69
TRAIL
CXCR3
NKG2D
71.7±17.5
4.5±1.8
26.5±6.4
5.4±0.8
104.3±24.5
6.7±0.6
27.8±3.2
19.5±1.6
46.6±26.3
3.1±1.7
21.5±3.5
4.4±0.2
77.1±23.0
5.2±2.0
26.6±4.5
17.5±1.4

Figure S10

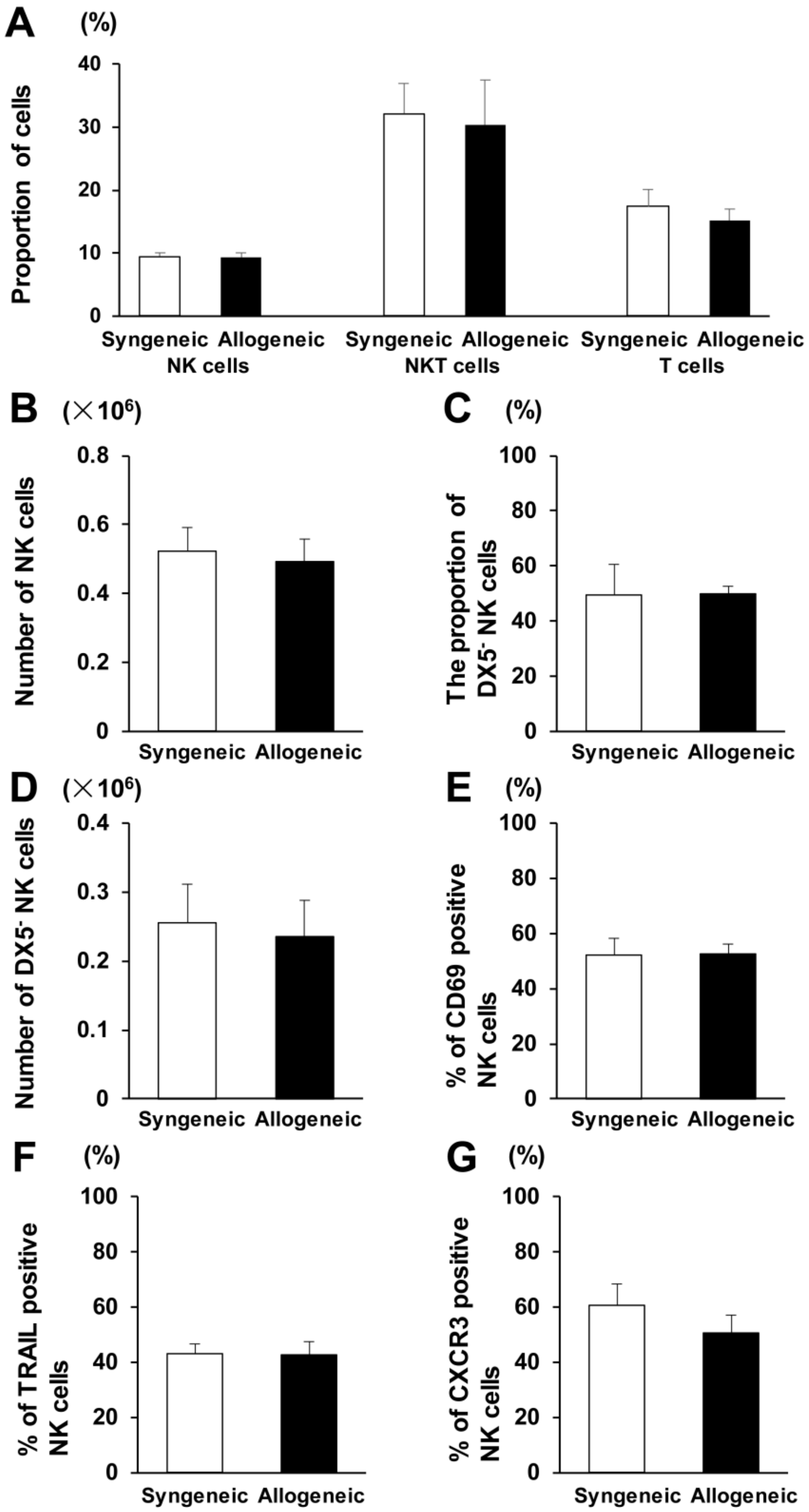

A
(%)
Proportion of cells
40
30
20
10
0
Syngeneic Allogeneic
NK cells
Syngeneic Allogeneic
NKT cells
Syngeneic Allogeneic
T cells
B
(×10⁶)
Number of NK cells
0.8
0.6
0.4
0.2
0
Syngeneic Allogeneic
C
(%)
The proportion of DX5⁻ NK cells
100
80
60
40
20
0
Syngeneic Allogeneic
D
(×10⁶)
Number of DX5⁻ NK cells
0.4
0.3
0.2
0.1
0
Syngeneic Allogeneic
E
(%)
% of CD69 positive NK cells
100
80
60
40
20
0
Syngeneic Allogeneic
F
(%)
% of TRAIL positive NK cells
100
80
60
40
20
0
Syngeneic Allogeneic
G
(%)
% of CXCR3 positive NK cells
100
80
60
40
20
0
Syngeneic Allogeneic

Figure S11

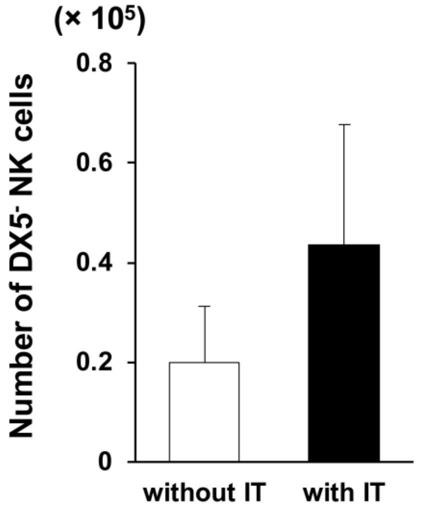

Figure S12

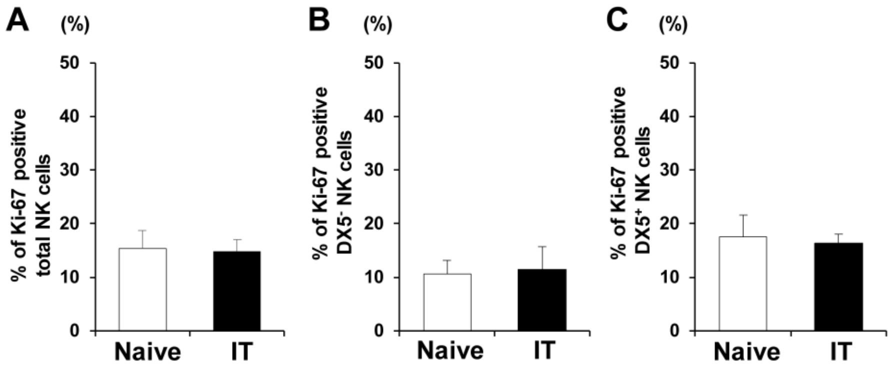

A
(%)
50
40
30
20
10
0
% of Ki-67 positive total NK cells
Naive
IT
B
(%)
% of Ki-67 positive DX5⁻ NK cells
Naive
IT
C
(%)
% of Ki-67 positive DX5⁺ NK cells
Naive
IT

Figure S13

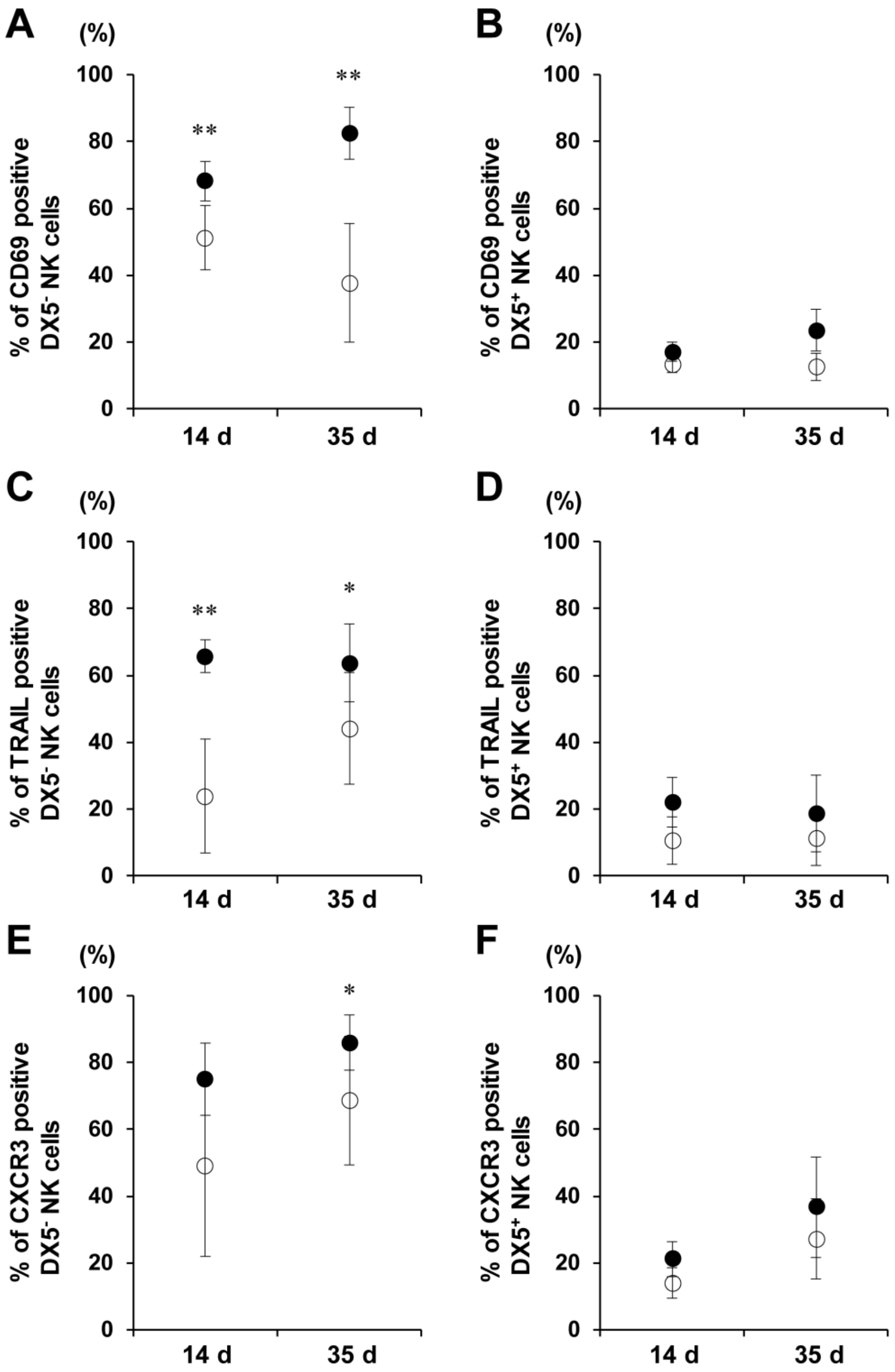

A
(%)
100
80
60
40
20
0
% of CD69 positive DX5⁻ NK cells
**
**
14 d
35 d
B
(%)
% of CD69 positive DX5⁺ NK cells
14 d
35 d
C
(%)
% of TRAIL positive DX5⁻ NK cells
**
*
14 d
35 d
D
(%)
% of TRAIL positive DX5⁺ NK cells
14 d
35 d
E
(%)
% of CXCR3 positive DX5⁻ NK cells
*
14 d
35 d
F
(%)
% of CXCR3 positive DX5⁺ NK cells
14 d
35 d

Figure S14

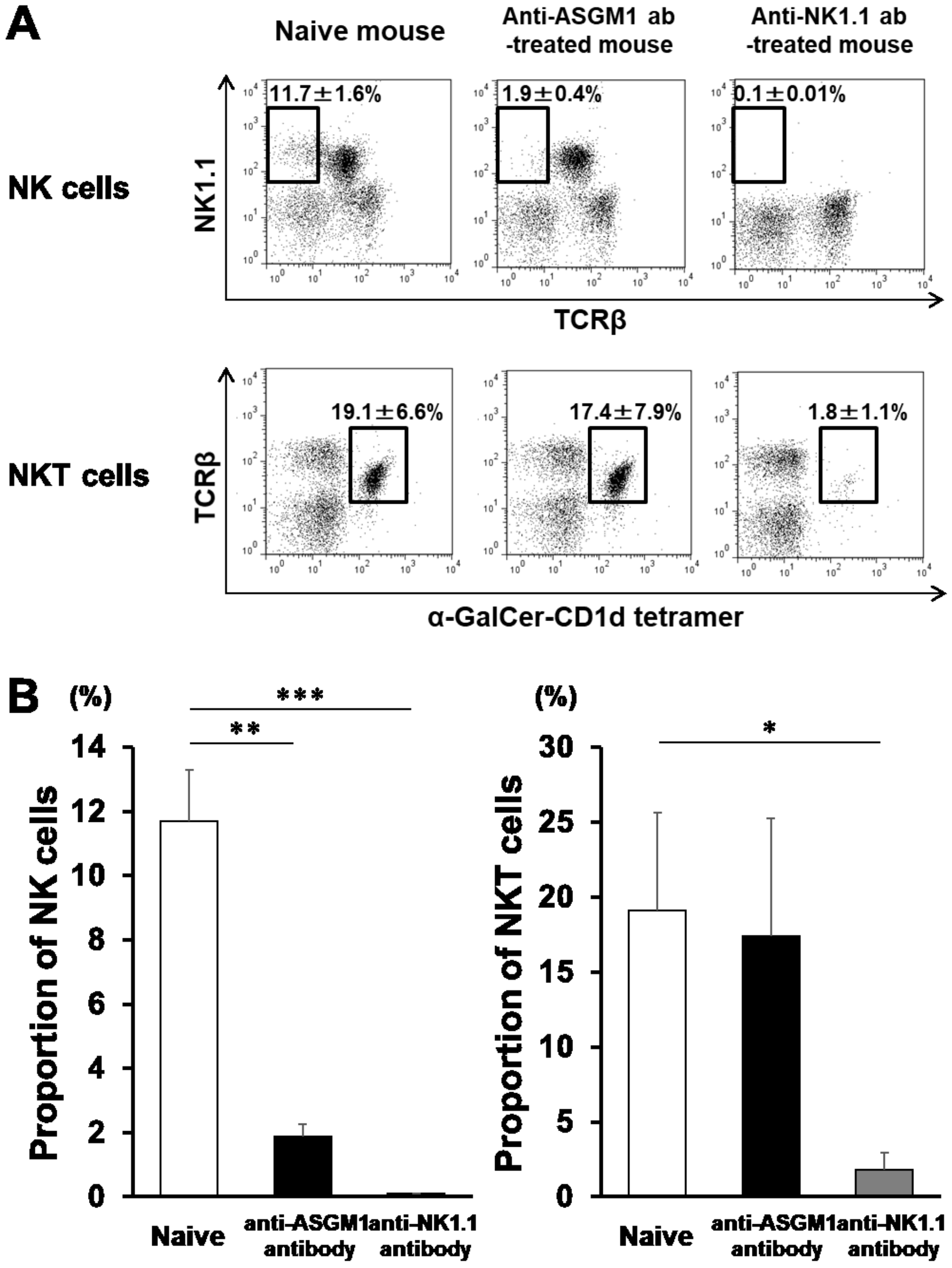

A
Naive mouse
Anti-ASGM1 ab -treated mouse
Anti-NK1.1 ab -treated mouse
NK cells
NK1.1
11.7±1.6%
1.9±0.4%
0.1±0.01%
TCRβ
NKT cells
TCRβ
19.1±6.6%
17.4±7.9%
1.8±1.1%
α-GalCer-CD1d tetramer
B
(%)
***
**
Proportion of NK cells
Naive
anti-ASGM1 antibody
anti-NK1.1 antibody
(%)
*
Proportion of NKT cells
Naive
anti-ASGM1 antibody
anti-NK1.1 antibody

Figure S15

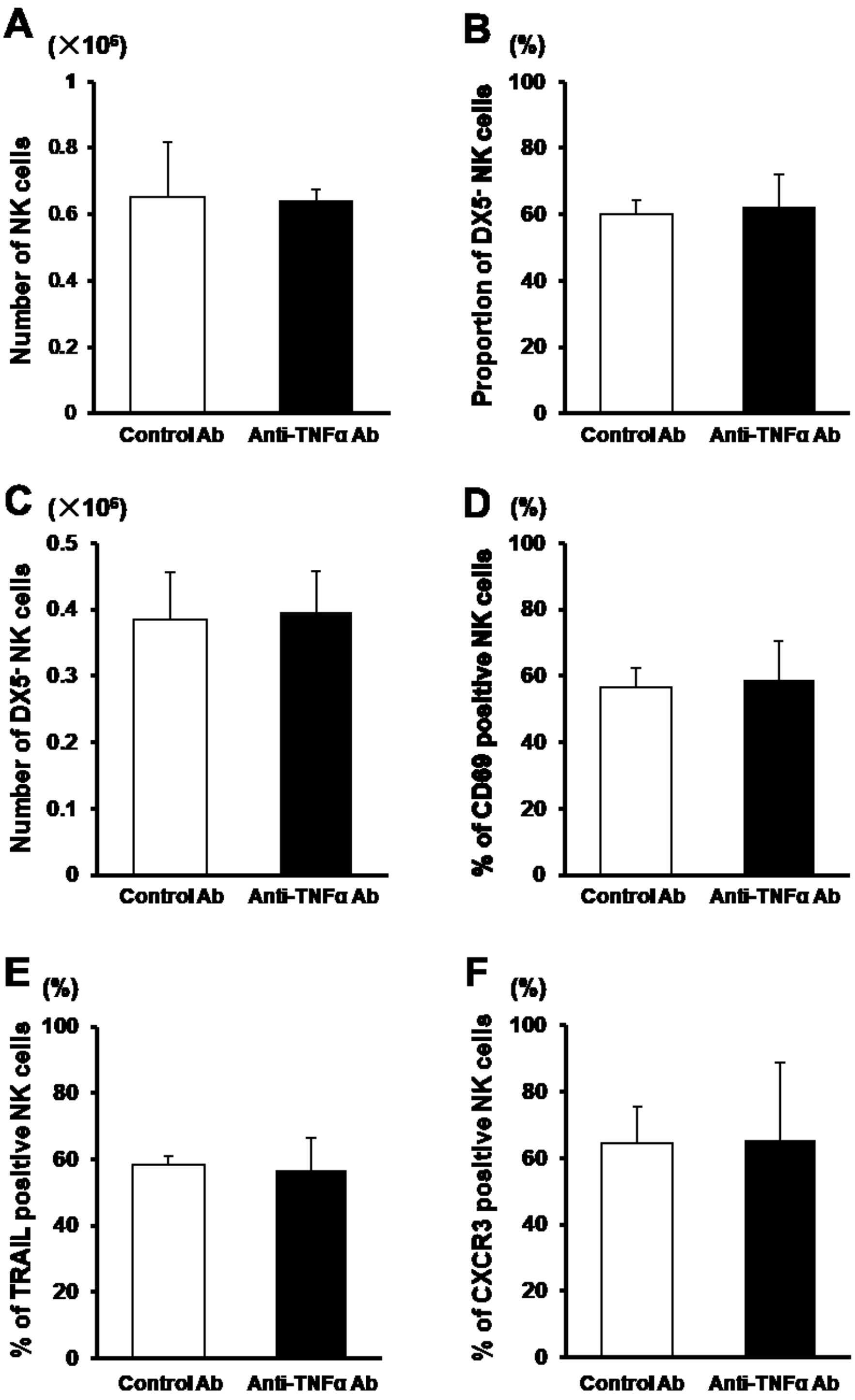

A
(×10⁶)
Number of NK cells
1
0.8
0.6
0.4
0.2
0
Control Ab
Anti-TNFα Ab
B
(%)
Proportion of DX5⁻ NK cells
100
80
60
40
20
0
Control Ab
Anti-TNFα Ab
C
(×10⁶)
Number of DX5⁻ NK cells
0.5
0.4
0.3
0.2
0.1
0
Control Ab
Anti-TNFα Ab
D
(%)
% of CD69 positive NK cells
100
80
60
40
20
0
Control Ab
Anti-TNFα Ab
E
(%)
% of TRAIL positive NK cells
100
80
60
40
20
0
Control Ab
Anti-TNFα Ab
F
(%)
% of CXCR3 positive NK cells
100
80
60
40
20
0
Control Ab
Anti-TNFα Ab
